# CD39 is an antibody-secreting B-cell marker that modulates germinal center and antibody responses during infection

**DOI:** 10.3389/fimmu.2025.1547929

**Published:** 2025-10-20

**Authors:** Laura Almada, Yamila N. Gazzoni, Cristian G. Beccaria, Facundo Fiocca Vernengo, Santiago Boccardo, Melisa Gorosito Serrán, Apurwa Trivedi, Carola G. Vinuesa, Simon C. Robson, Eva V. Acosta Rodríguez, Mauro Gaya, Carolina L. Montes, Adriana Gruppi

**Affiliations:** ^1^ Departamento de Bioquímica Clínica, Facultad de Ciencias Químicas (FCQ), Universidad Nacional de Córdoba, Córdoba, Argentina; ^2^ Centro de Investigaciones en Bioquímica Clínica e Inmunología (CIBICI - CONICET), Córdoba, Argentina; ^3^ Centre d’Immunologie de Marseille-Luminy (CIML), Aix Marseille Université, Institut national de la santé et de la recherche médicale (INSERM), Centre national de la recherche scientifique (CNRS),, Marseille, France; ^4^ John Curtin School of Medical Research (JCSMR), Canberra, ACT, Australia; ^5^ The Francis Crick Institute, London, United Kingdom; ^6^ Center for Inflammation Research, Department of Anesthesia, Critical Care and Pain Medicine, Beth Israel Deaconess Medical Center, Harvard Medical School, Boston, MA, United States

**Keywords:** ADO, *Trypanosoma cruzi*, influenza virus, antibody secreting cell (ASC), germinal center (GC), plasmablast, plasma cell (PC)

## Abstract

CD39 is an ectoenzyme in immune cells that regulates purinergic signaling by converting extracellular ATP into adenosine (ADO). Although first described on EBV-transformed B cells, CD39’s role in humoral immunity remains unclear. Using murine infection models and human samples, we confirm and extend previous findings showing that high CD39 expression identifies antibody-secreting cells (ASC) across differentiation stages, including ASC derived from memory B cells, and in various tissues, regardless of the infection phase. CD39 was resistant to enzymatic digestion, facilitating ASC identification in processed tissues. We found that while CD39 was not essential for B-cell differentiation into ASC, it remained functionally active as an ectoenzyme. ASC as well as germinal center (GC) B cells expressed ADO receptors, making them responsive to ADO signaling. Consistently, systemic ADO administration impaired GC reactions without altering the ASC number in infected mice. However, *in vitro*, ADO reduces antibody production both in ASC and in B cells undergoing differentiation and also impairs the differentiation of activated B cells. Finally, B cell–specific CD39 deficiency increased GC B-cell frequencies in infected mice, likely due to reduced ADO levels. These findings highlight the relevance of the purinergic pathway in B-cell biology.

## Introduction

Antibody-secreting cells (ASC) play a crucial role in protecting against pathogens through their particular capacity for immunoglobulin production. ASC represent the final stage of B-cell differentiation and are conventionally classified as either plasmablasts (PB), which are proliferative and short-lived, or the terminally differentiated subpopulation, PC, which are typically non-proliferative, produce larger amounts of antibody and can persist for extended periods (1). Besides this classification according to differentiation stage, ASC are heterogeneous containing diverse subpopulations with different capabilities regarding cytokine production, antigen presentation, and regulatory function (2–5). ASC exhibit remarkable versatility, showcasing an extraordinary ability to modulate immune responses independently of antibody production. Indeed, subsets of ASC are highly effective at regulating immune reactions through IL-10 or IL-35 production, crucial for maintaining tissue and immune homeostasis. Moreover, ASC roles are not limited to protection, as they also play a significant role in autoimmune diseases and can undergo malignant transformation as evidenced in multiple myeloma (5–7).

Most of our knowledge about B-cell responses and ASC generation comes from experimental models using non-replicating antigens with adjuvants. In these models, the “canonical” response involves BCR signaling and T-cell priming at the T:B borders of secondary lymphoid tissues. This leads to a rapid extrafollicular PB response that generates short-lived plasma cells *in situ*, along with germinal center (GC) responses that can last for a few weeks (8). PB secrete antibodies while dividing and then differentiate into non-dividing PC that produce larger amounts of antibodies. Their numbers peak 4–6 days after immunization, rapidly declining thereafter. Concurrently, some primed T and B cells migrate into B-cell follicles, where they continue to proliferate and commit to the GC fate. The GC ultimately serves as the primary source of long-lived ASC, also known as long-lived plasma cells (LLPCs), which may migrate to the bone marrow (BM). ASC exhibit phenotypic and genotypic differences depending on their environment, including variations in surface markers and chemokine receptors. Notably, some surface markers correlated with ASC lifespan (9).

Due to their heterogeneity, and the loss of many canonical B-cell markers, ASC cannot necessarily be identified by few cell surface markers. ASC are classically defined in humans by the increased expression of CD38, CD27, and CD138 (Syndecan-1), whereas in mice, CD138 is routinely used in conjunction with the downregulation of B220 and increased Blimp-1 and IRF-4 expression (10). The strategies developed to identify mouse ASC also include the expression of cell surface proteins such as Sca-1 (Ly6A/E), CD267 (TACI or TNFRSF13B), or CD98 in combination with CD138 (11, 12). Recently, 12 new surface molecules have been suggested to identify mouse and human ASC. Among these, CD39 (NTPDase1), CD81 (TAPA-1), and CD130 (gp130 or Oncostatin M receptor) have been proposed as useful protein markers for ASC identification (13). The identification of these markers was performed by evaluating ASC from normal animals, as well as in mice and humans under autoimmune conditions such as those present in Lupus (13). Therefore, it is important to evaluate whether the expression of these molecules occurs in distinct types of ASC from different tissues under inflammatory conditions other than autoimmunity or vaccination, such as in infections.

CD39 is an ectonucleoside triphosphate diphosphohydrolase-1 (ENTPD1) that catalyzes the hydrolysis of extracellular ATP (eATP) and ADP to AMP. By working in tandem with CD73, the ecto-5-nucleotidase (E5NT) converts AMP to adenosine (ADO), a nucleoside with pleiotropic functions including immunosuppression (14). CD39 and CD73 are known to be expressed on human regulatory B cells (15, 16). CD39^hi^ PB increase in mice spleen and generate high levels of extracellular ADO, as noted in a model of sepsis (17). This nucleoside impairs macrophage bactericidal activity via binding to the ADO receptor (ADOR) A2a and enhances interleukin (IL)-10 production, further demonstrating the regulatory function of CD39^hi^PB (17). While the function of CD39 in B lineage cells has mainly been linked to a regulatory role, the potential effects of the products of its enzymatic activity on the B cell itself remain largely unexplored. It has been reported that ADO contributes to class switch recombination (CSR) in human naive and IgM^+^ memory B cells (18). However, it is currently unknown how CD39 and the catalytic pathways toward extracellular ADO generation control the magnitude and quality of B-cell responses *in vivo*.

In this work, we investigated the expression and function of CD39 within the B-cell compartment, particularly focusing on ASC. These studies were conducted across experimental mouse models of infection and in a clinical setting. Our findings confirmed high expression of CD39 as an unequivocal ASC marker located in different tissues/cellular environments. Moreover, we identified a novel role for CD39 and ADO, an indirect product of CD39 activity, in shaping the magnitude of antibody response, contributing to the regulation of GC reaction and immunoglobulins production.

## Results

### ASC at different phases of *T. cruzi* infection exhibit elevated CD39 expression levels

Prior evidence suggested that CD39 was a potential marker for PC (13). Therefore, we investigated the expression of CD39 across different ASC populations under physiological conditions and in infection settings. To achieve this, we studied various experimental models of infections in mice, as well as SARS-CoV-2 infection in humans.

**Figure 1:**
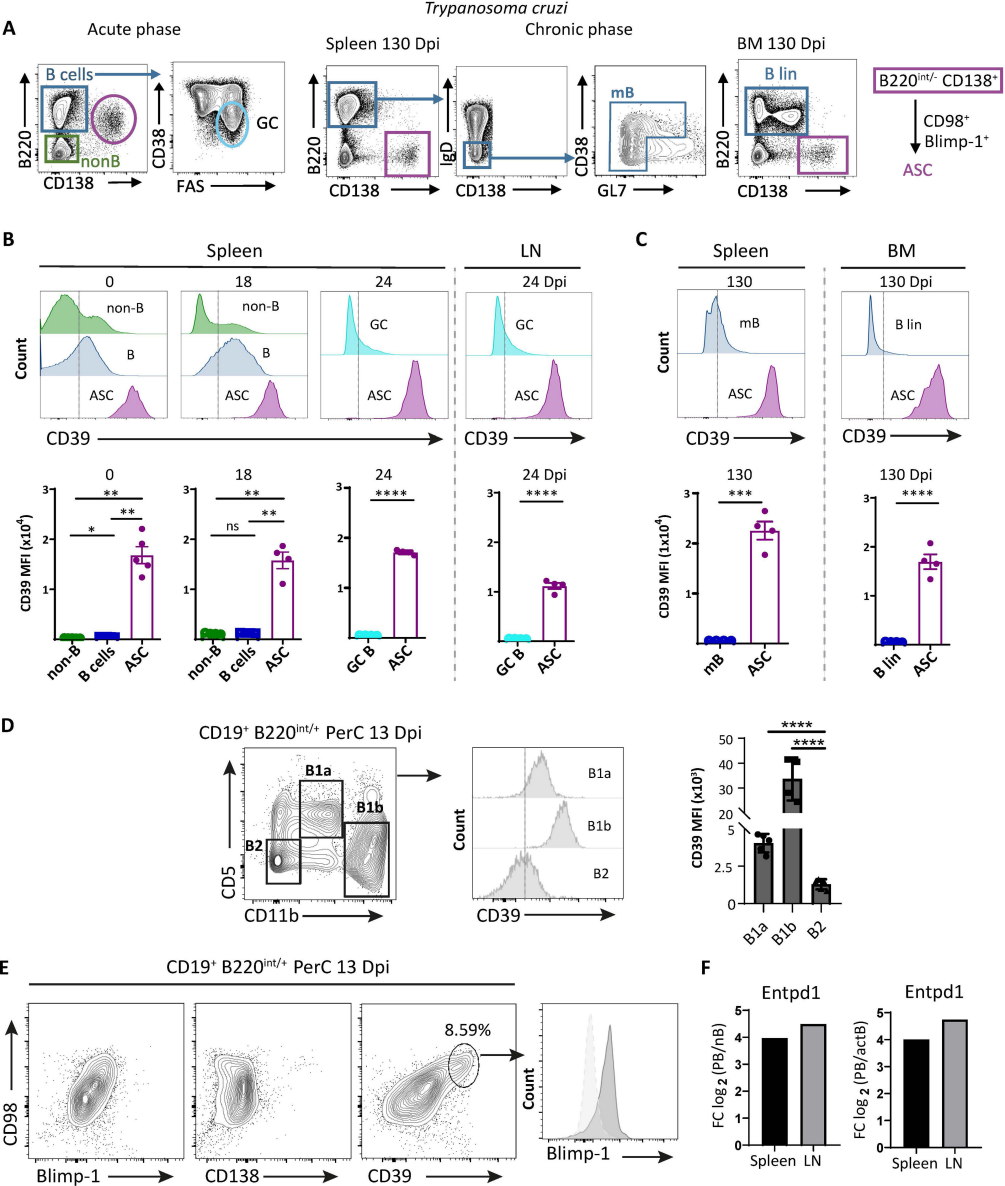
ASC from different lymphoid tissues of *T. cruzi*-infected mice expressed high levels of CD39. **(A)** Gating strategies for the identification of ASC and GC B cells during the acute phase of infection, and ASC and memory B (mB) cells in the spleen of chronically infected mice. The strategy used to identify ASC and Blin (B lineage, B220^+^CD138^−^ non-antibody secreting B cells) cells in the BM of chronically infected mice is also shown. The purple box highlights a representative dot plot indicating that ASC from all stages and tissues analyzed express CD98. **(B)** Representative histograms and statistical analysis showing the expression of CD39 on non-B cells, B cells, and ASC in spleen from uninfected C57BL/6 mice (day 0 post-infection, 0 Dpi) and *T. cruzi* infected mice obtained at 18 Dpi. CD39 expression is also shown in GC B cells and ASC from the spleen and LN obtained at 24 Dpi. The Fluorescence Minus One control (FMO) is indicated with a dashed line. **(C)** Representative histograms and statistical analysis showing the expression of CD39 in mB and ASC from spleen and Blin and ASC from BM at 130 Dpi (FMO indicated as in **A**). **(D)** Gating strategy for the identification of B1a, B1b, and conventional B2 cells in PerC from 13-Dpi *T cruzi*-infected mice. Representative histograms and statistical analysis showing the expression of CD39 on B1a, B1b, and conventional B2 cells from peritoneal cavity (PerC) of 13 Dpi-infected mice. In **(B, D)** Bars represent the mean ± standard deviations of the mean fluorescence intensity (MFI) of CD39 expression in the aforementioned cell populations. Dots represent the number of mice. **(E)** Representative contour plots of CD98 vs. Blimp-1, CD138, and CD39 of gated CD19^+^B220^+/int^ cells and representative histogram of Blimp-1 expression in CD39^+^CD98^+^ cells. All flow cytometry results are representative of at least three independent experiments, with n ≥ 4. **(F)** Naive B cells (nB), activated B cells (actB), and ASC from spleen and LN were purified by cell sorting at 20 Dpi and analyzed by bulk RNA-seq. Each bar corresponds to the relative expression level of Entpd1 (CD39 coding gene) in PB compared with nB and actB (x-axis). The y-axis represents the log2 of expression fold-change (Log2FC) for each gene. Positive values indicate upregulated genes in PB samples compared with nB or actB. The Wald test was used to generate p-values and log2 fold changes. Bars graphed genes had an adjusted p-value <0.05 and absolute log2 fold change >1 (differentially expressed genes). *p<0.05, **p<0.01, ***p<0.001, ****p < 0.0001.

Using the *T. cruzi* infection model, we evaluated the expression of CD39 by flow cytometry in cells belonging to the B-cell compartment, such as GC and memory B cells and ASC, and in non-B cells from the spleen and inguinal lymph nodes (LN) of infected mice at the acute phase (18–28 days post-infection, Dpi) and in the spleen and BM at the chronic phase (130 Dpi) of the infection (see gating strategies in **Figure 1A**, **Supplementary Figure S1**).

In **Figure 1B**, representative histograms of CD39 expression as well as the mean fluorescence intensity (MFI) of CD39 in the different cell types analyzed are presented (gating strategy shown in **Figure 1A** and in **Supplementary Figure S1A**). The small ASC population (B220^int^CD138^+^CD98^+^) present in the spleen of non-infected WT mice (0 Dpi) exhibited a higher expression of CD39 in comparison with B cells (B220^+^CD138^neg^) and non-B cells (B220^neg^). Splenic ASC, present in the acute phase of *T. cruzi* infection (at 18 Dpi), identified as PB (B220^int^CD138^+^CD98^+^Ki67^+^Blimp-1^+^, see **Supplementary Figure S1A**) also had high expression of CD39 (**Figure 1B**). At 24 Dpi, a time point when GC are established in infected mice (19), we observed that the ASC population in the spleen and LN (**Figure 1B**) expressed higher levels of CD39 compared with GC B cells (defined as B220^+^CD38^−^FAS^+^, see **Supplementary Figure S1A**), of which only a small fraction expressed low levels of CD39. ASC from spleen and BM, identified as PC (B220^neg^CD138^+^CD98^+^Ki67^neg^Blimp-1^+^, gating strategy in **Supplementary Figures S1B, C**), from chronic infected mice (130 Dpi) also exhibited a higher CD39 expression than splenic memory B cells (mB, defined as B220^+^CD138^neg^IgD^neg^non-GC, see **Supplementary Figure S1B**) (**Figures 1A, C**) and B lineage cells (B lin, B220^+^CD138^neg/low^) from BM. B lin in BM refers to non-antibody-secreting B cells.

It has been reported that peritoneal B1 cells, which spontaneously secrete IgM and IgG_3_ and are classified as natural ASC, have a higher CD39 expression, determined as MFI, than peritoneal and splenic B2 cells (20). Based on this, we evaluated the expression of CD39 in B cells from peritoneal cavity (PerC) of *T. cruzi*-infected mice at 13 Dpi (gating strategy shown in **Figure 1D**, **Supplementary Figure S1E**). As expected (20), peritoneal B1a and B1b cells expressed significant higher levels of CD39 compared with conventional peritoneal B2 cells (**Figure 1D**). Using CD39 as a marker, we were able to clearly identify an ASC population (CD39^+^CD98^+^ cells expressing Blimp-1^+^ within the CD19^+^B220^+/int^ gate), which was not detectable when using anti-CD138 (**Figure 1E**). Although ASC in the PerC of *T. cruzi*-infected mice lack CD138 expression (21), their identification by flow cytometry is enabled by the use of anti-CD39. We confirmed that the ASC identified initially as B220^int^CD138^+^ were double-positive for both CD98 and CD39 (**Supplementary Figure S1D** and schematically shown in **Figure 1A**).

By RNA sequencing, we evaluated sorted purified PB (B220^int^CD138^+^CD98^+^), naïve (B220^+^IgD^+^CD138^neg^), and activated B cells (B220^+^IgD^neg^CD138^neg^) from the spleen and inguinal LN from *T. cruzi*-infected mice at 20 Dpi. PB were selected for the analysis as they represent the major ASC population (99% of the ASC) at this stage of the infection (19). We detected a significant upregulation of the Entpd1 gene, which encodes CD39, expression in ASC when compared with both naive (**Figure 1F**, left graph) and activated B cells (**Figure 1F**, right graph) across both tissues.

### ASC expressing elevated CD39 are also present in bacterial and viral infections

To determine whether elevated CD39 expression detected in ASC from *T. cruzi*-infected mice is a shared feature across different infections, we examined CD39 expression levels in ASC generated during both bacterial and viral infections in mice.

**Figure 2:**
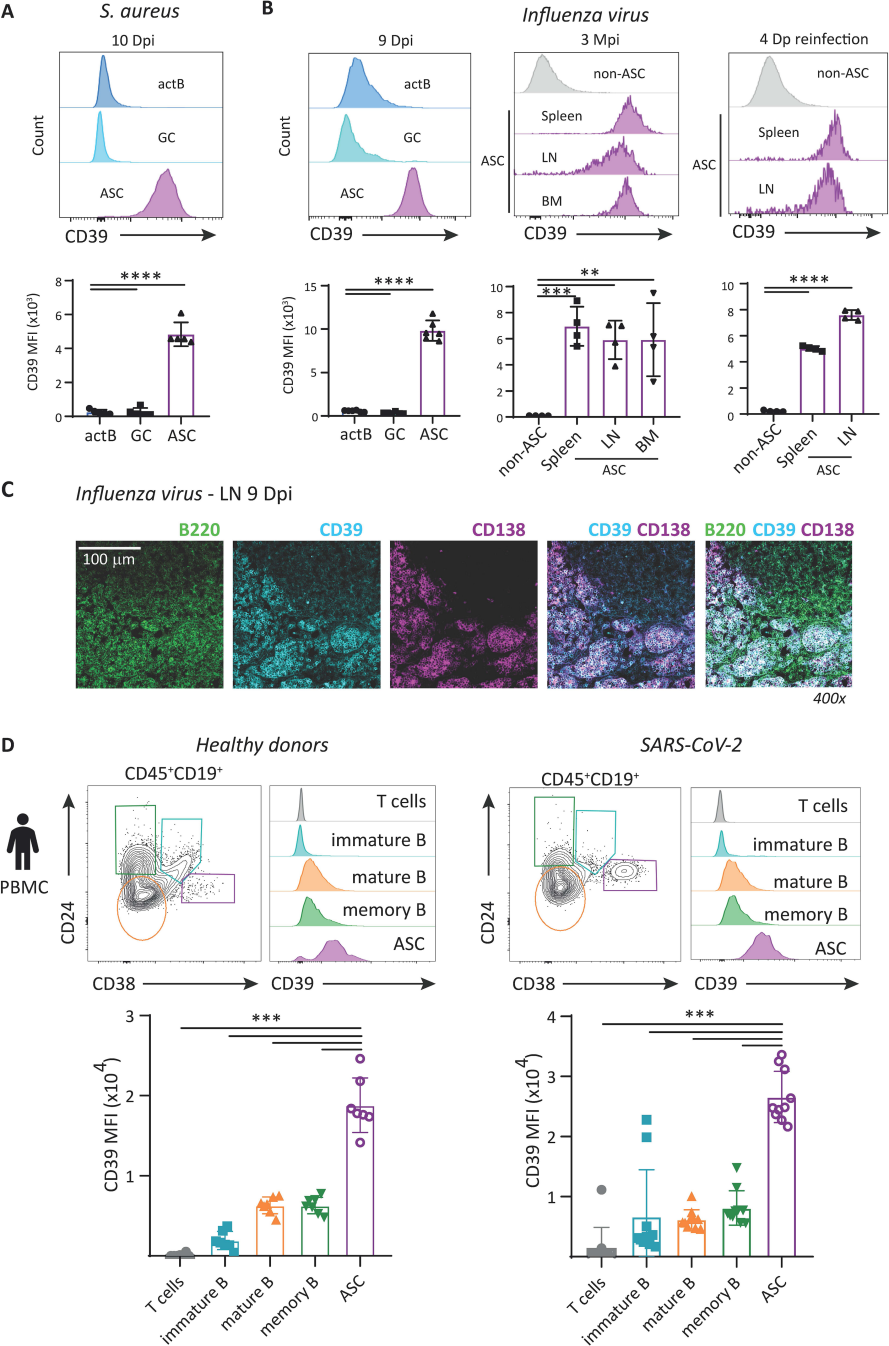
The ASC in bacterial and viral infections expressed high levels of CD39. **(A)** Representative histograms and statistical analysis showing the expression of CD39 on **(A)** activated (actB), GC B cells, and ASC of the ear-draining LN obtained at 10 Dpi from mice intradermally infected with *S. aureus*. **(B)** act, GC B cells, and ASC of mediastinal LN obtained at 9 Dpi from mice intranasally infected with *Influenza virus* PR8 strain, and ASC present in the spleen, LN, and BM from *Influenza* infected mice at 3 months post-infection (Mpi) and on ASC from the spleen and LN obtained at 4 days post-*Influenza* reinfection (Dp reinfection) and on splenic non-ASC B cells. All flow cytometry data came from at least two independent experiments, with n ≥ 4. **(C)** Immunofluorescence of a section of mediastinal LN obtained at 9 Dpi from mice intranasally infected with *Influenza* stained with anti-B220, -CD39, and -CD138; overlapping images of the staining are also shown. **(D)** Representative contour plot of CD24 vs. CD38 expression in Live CD45^+^ CD19^+^ PBMC from healthy donors and SARS-CoV-2-infected individuals (COVID-19). The plots allow for the identification of immature, mature, memory, and ASC. Representative histograms and statistical analysis of CD39 expression on T cells and immature, mature, and memory B cells, and ASC of PBMC from healthy donors (left, n=8) and COVID-19 patient (right, n=10). ***p<0.001 Statistical analysis corresponds to the comparison of CD39 expression in ASC relative to each of the other populations. **p<0.01, ****p < 0.0001.

By flow cytometry, comparison of CD39 expression shows that following *S. aureus* infection, the ASC within the submaxillary draining LN, obtained at 10 Dpi, expressed significantly increased levels of CD39 in contrast to activated and GC B cells (**Figure 2A**, **Supplementary Figure S2A**).

Additionally, ASC from the mediastinal LN of *Influenza virus H1N1* PR8-infected mice, obtained at 9 Dpi, displayed a significantly higher expression of CD39 in comparison with activated and GC B cells (**Figure 2B**, left panel). The ASC present in the spleen, LN, and BM of mice after 3 months of *Influenza virus-*PR8 strain infection also showed a high expression of CD39 in comparison with splenic non-ASC B cells (CD19^+^CD138^neg^) (**Figure 2B**, middle panel), indicating that, regardless of the phase of infection, all ASC expressed significantly higher levels of CD39, as previously shown for ASC present in *T. cruzi*-infected mice.

To evaluate CD39 expression in ASC generated upon secondary responses, mice recovered from initial infection with *Influenza virus H1N1* PR8 were rechallenged with influenza H3N2 X31 strain. The ASC were evaluated across various tissues. After 4 days, post-second *Influenza* infection, we noted that the ASC generated in the spleen and mediastinal LN (**Figure 2B**, right panel) all exhibited higher levels of CD39 in comparison with splenic CD19^+^CD138^neg^ cells (non-ASC B cells). In the lung, we observed that the ASC population (identified as CD98^+^IgD^−^CD19^+^non-GC) also expressed high levels of CD39 (**Supplementary Figure S2C**). Consistent with previous reports (22), a significant proportion of lung ASC were IgA+ (**Supplementary Figure S2C**). In conclusion, on each day that CD39 expression was analyzed, ASC expressed higher levels of CD39 than the other cells present in the same infected animal.

Immunofluorescence analysis of LN sections from 9 Dpi *Influenza virus*-infected mice showed a similar pattern of CD138^+^ (purple) and CD39^+^ (cyan) cells, and co-expression of both markers (white) on ASC, confirming the CD39 expression on ASC. B220 staining (green) was used for the identification of B lineage cells. The image, with the three overlaid fluorochromes, highlights the B-cell area and the ASC expressing CD39 and CD138 (**Figure 2C**).

We next determined whether the high expression of CD39 in ASC is also present in human cells. We examined CD39 levels on various subsets within B-cell lineage, as well as on T cells, in PBMC of healthy donors and from individuals infected with SARS-CoV-2. **Figure 2D** shows representative dot plots of CD24 vs. CD38 expression within gated CD19^+^ lymphocytes, which allows for the identification of distinct B-cell subsets. The collected data revealed that circulating mature and memory B cells from healthy donors and SARS-CoV-2-infected patients exhibited markedly elevated CD39 levels, when compared with immature B and also to T cells. Notably, the highest expression of CD39 was seen in the ASC population in healthy controls and COVID-19 patients.

Taken together, all these findings demonstrate that in infections caused by different types of pathogens, the ASC subpopulations exhibited a consistently elevated CD39 expression. These results were observed across acute and chronic stages of infection in both murine models and human.

To validate our observations in infection models and corroborate previously reported data (13), we examined ASC from SRBC-HEL immunized mice and several autoimmune models (Fas-Lpr, Sanroque, and Trex), which consistently expressed high levels of CD39 (**Supplementary Figure S3**).

### CD39 expression is dispensable for immunoglobulin class switching

Taking into account that nearly 100% of ASC express high levels of CD39, we hypothesized that CD39 functions extend beyond its traditionally recognized regulatory role, typically attributed to a “subset” of ASC (7, 15). To evaluate the biological relevance of CD39 in the ASC, we took advantage of CD39 knockout (CD39^−/−^) mice. CD39^−/−^ mice were infected with *T. cruzi* and the frequency of ASC in the spleen and inguinal LN was examined. Spleen and LN were selected for the study because they undergo marked hypertrophy during the acute phase of *T. cruzi* infection, and where the primary immune response occurs.

**Figure 3:**
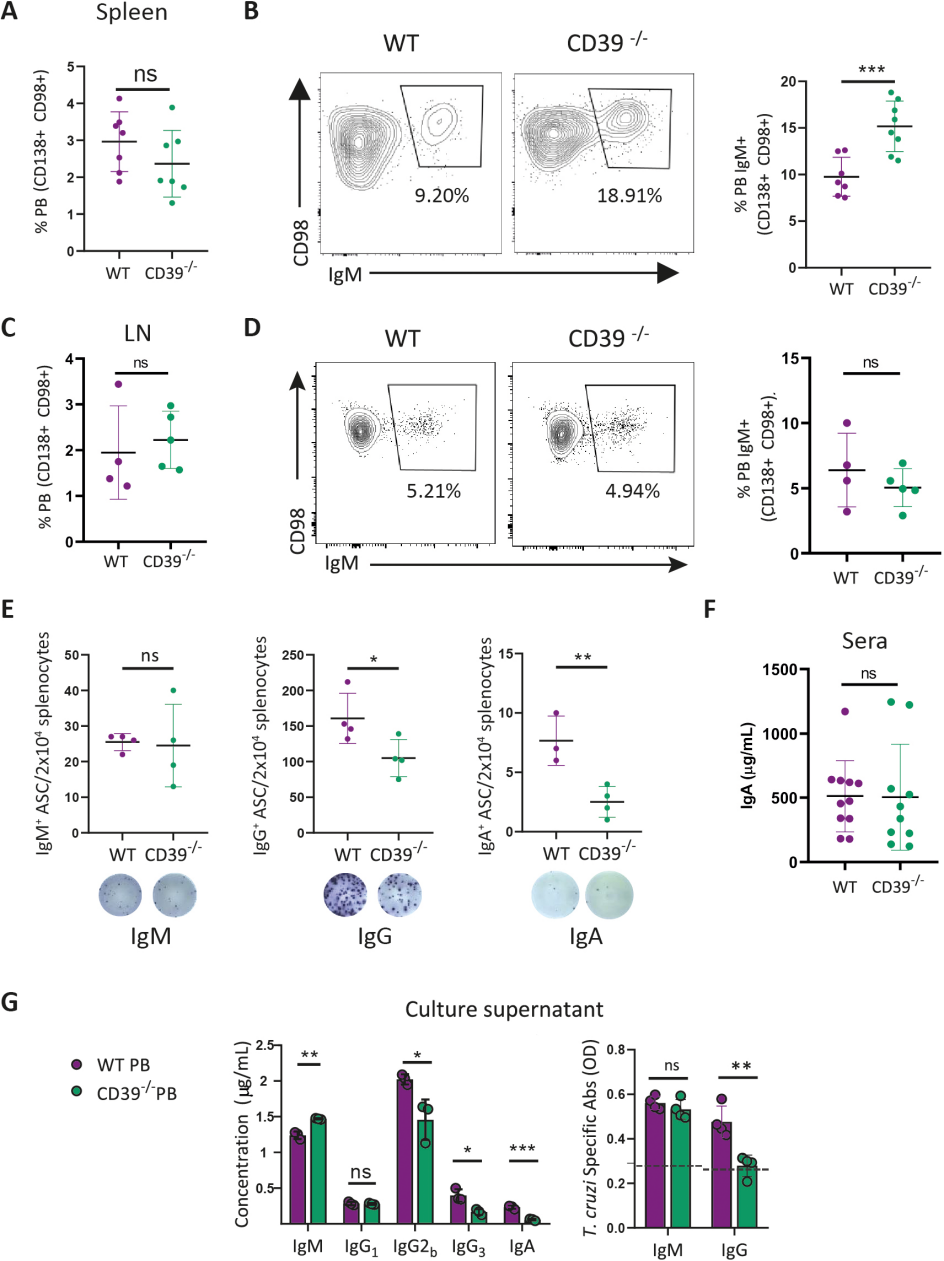
CD39 expression was dispensable for ASC generation and immunoglobulin class-switch. Dot plot with statistical analysis showing the frequency of PB (B220^int^CD138^+^CD98^+^ki-67^+^Blimp-1^+^) within the B-cell compartment (B220^+^ and CD138^+^B220^int^ cells) in **(A)** the spleen or **(C)** inguinal lymph node (LN) of WT and CD39^−/−^ mice infected with *T. cruzi* obtained at 18 Dpi. **(B)** Representative contour plots and statistical analysis showing the frequency of IgM^+^ within CD138^+^CD98^+^ PB in the **(B)** spleen or **(D)** inguinal LN. **(E)** Numbers of IgM, IgG, or IgA producing ASC in 2 × 10^4^ splenocytes from 18 Dpi-*T. cruzi*-infected WT and CD39^−/−^ mice obtained by ELISpots. Photographs are representative of the corresponding ELISpot. **(F)** Total IgA serum concentration of *T. cruzi*-infected WT and CD39^−/−^ mice at 18 Dpi. **(G)** Total immunoglobulin production determined by LEGENDplex (left) and *T. cruzi*-specific IgM and IgG determined by ELISA (right) in the culture supernatant of sorted PB from the spleen obtained at 18 Dpi-*T. cruzi*-infected WT and CD39^−/−^ mice. Dot lines show the positive/negative limit. **(A-D)** are representative of three independent experiments with n≥3. **(F)** presents data from three independent experiments (n= 10-12). **(G)** is representative of two independent experiments with n ≥ 4. *p<0.05, **p<0.01, ***p<0.001.

On analysis of PB, the main ASC present at 18 Dpi, frequencies in the spleen and LN revealed that CD39 was dispensable for PB generation, as both *T. cruzi-*infected WT and CD39^−/−^ mice exhibited similar percentages of PB (**Figures 3A, C**). Interestingly, flow cytometry analysis showed a higher frequency of splenic IgM^+^ PB in CD39^−/−^ mice compared with controls (**Figure 3B**). When analyzing inguinal LN, unlike the spleen, the frequency of IgM^+^ PB in the inguinal LN was not affected by the absence of CD39 (**Figure 3D**). ELISpot analysis revealed no significant difference in the frequency of splenic IgM-secreting cells (IgM^+^ASC) between WT and CD39^−/−^ mice, suggesting that while there is an increased presence of splenic IgM^+^PB in CD39^−/−^ mice, this does not translate into a higher number of IgM-secreting cells (**Figure 3E**). Although CD39^−/−^ mice exhibited IgG^+^- and IgA^+^-secreting cells (IgG^+^ASC and IgA^+^ASC) in the spleen, their frequencies were significantly reduced compared with infected WT mice (**Figure 3E**). This indicates that while class switch recombination (CSR) occurs in the absence of CD39, the lack of this molecule reduces the frequency of splenic IgG- and IgA-producing cells. Then, we measured serum antibodies, focusing on IgA, as the differences in splenic IgA^+^ ASC in **Figure 3E**. We found similar concentrations of serum IgA in both infected CD39^−/−^ and WT mice (**Figure 3F**). Moreover, splenic PB, purified by cell sorting, from infected CD39^−/−^ mice secreted significantly lower amounts of IgG2b, IgG3, and IgA than PB from infected WT mice (**Figure 3G**). Consistent with the reduction in IgG2 and IgG3, we observed lower levels of parasite antigen-specific IgG in the culture supernatant of sorted splenic PB from *T. cruzi*-infected CD39^−/−^ mice compared with their counterparts from infected WT mice (**Figure 3G**).

Altogether, these data indicate that the absence of CD39 did not prevent Ig class switching, but it did reduce the frequency of splenic IgG and IgA-producing cells, suggesting that CD39 may, to some extent, influence the magnitude of the antibody response in specific tissues.

### ASC express a functional CD39 together with other molecules involved in the purinergic pathway

**Figure 4:**
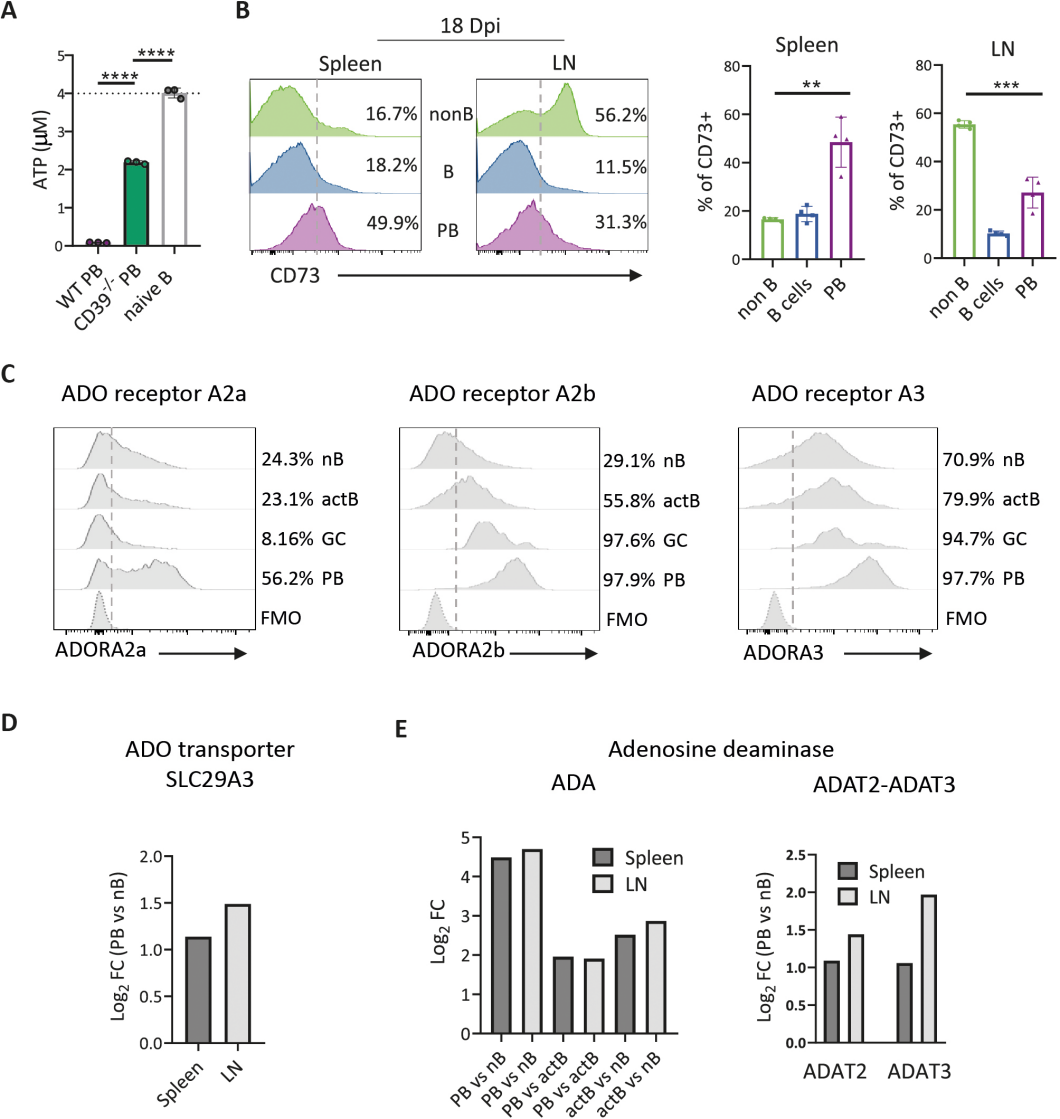
ASC expressed molecules involved in the ADO pathway. **(A)** ATP levels on culture supernatants from splenic PB purified from *T. cruzi*-infected WT or CD39^−/−^ mice cultured with exogenous ATP. Sorted naive B cells were used as control. **(B)** Representative histograms and statistical analysis of CD73 expression on non-B cells (B220^neg^), B cells (B220^+^CD138^neg^), and PB (B220^int^CD138^+^ CD98^+^Ki-67^+^) of the spleens and LN from *T. cruzi*-infected WT mice obtained at 18 Dpi. Numbers in each histogram indicate the frequency of CD73^+^ cells. **(C)** Representative histograms of ADO receptor expression (ADORA2a (left), ADORA2b (center), and ADORA3 (right)) on naive B cells (nB), activated B cells (actB non-GC), GC, and PB. N = 4. Numbers in each histogram indicate the frequency of positive cells **(D, E)** Naive B cells (nB, B220^+^CD138^neg^IgD^+^), activated B cells (actB, B220^+^IgD^neg^CD138^neg^), and PB from spleen and LN from 20 Dpi-*T. cruzi*-infected mice were purified by cell sorting and analyzed by bulk RNA-seq. Bar graphs show **(D)** SL29A3 (nucleoside transporter coding gene) relative expression levels in PB compared with nB and **(E)** adenosine deaminase (ADA) and ADAT2-ADAT3 complex mRNA relative expression levels in PB compared with nB and actB (x-axis). An increase in the expression of mRNA encoding for SL29A3 or ADA was depicted as log_2_Fold Change (FC) (y-axis). Positive values indicate upregulated genes in PB samples compared with nB or actB. The Wald test was used to generate p-values and log_2_FC. Bars graphed genes had an adjusted p-value < 0.05 and absolute log_2_FC > 1 (differentially expressed genes), n = 3 for each group. **(A-C)** Are representative of three independent experiments with n ≥ 3, analyzed with a T-test. **p<0.01, ***p<0.001, ****p < 0.0001.

To determine whether CD39 expression on ASC has ectonucleotidase activity, we performed *in vitro* ATP hydrolysis assays using splenic PB obtained from *T. cruzi*-infected mice at 18 Dpi, isolated through cell sorting. Our findings revealed a significant decrease in eATP concentration in the supernatants of purified PB cultured with exogenously added ATP when compared with the concentration observed in the supernatant of naive B cells cultured under the same condition. The ATP hydrolysis was diminished in the supernatants of splenic PB from *T. cruzi*-infected CD39^−/−^ mice (**Figure 4A**), indicating that PB scavenged ATP, by large part through the involvement of CD39, thus underscoring the functional activity of CD39 in mediating ATP hydrolysis in ASC.

Although CD39 can signal intracellularly via its N-terminus binding to RanBPM (23), we focused specifically in its function mediated through the generation of extracellular ADO (24, 25). Next, we evaluated the expression of molecules involved in the CD39/CD73/ADO metabolic pathway in ASC and other lymphoid cells from *T. cruzi*-infected mice. As previously described (25), we observed that CD73 was expressed in a low percentage of splenic B and non-B cells and approximately half of the non-B-cell population present in LN (**Figure 4B**). Unlike CD39, CD73 was not uniformly present in the entire population of PB (the ASC at the acute phase of *T. cruzi* infection), but almost 50% of splenic PB and 30% of inguinal LN PB expressed CD73 (**Figure 4B**, bar graphs). To evaluate possible ADO–ASC interactions we examined ADO receptors (ADOR) and transporters. Flow cytometry analysis revealed that a higher frequency of PB expressed ADORA2a compared with naïve, activated, or GC B cells (**Figure 4C**). Interestingly, **Figure 4C** also shows that a high percentage of activated B cells, as well as nearly all GC B cells and PB, expressed ADORA2b and ADORA3, suggesting that PB and other B-cell subsets can be targets of ADO.

PB from spleen and LN also showed an upregulation of the gene encoding the nucleoside transporter SLC29A3, a high-affinity ADO transporter (26), when compared with naive B-cell counterparts as determined by RNA-seq analysis (**Figure 4D**). Additionally, we observed changes in the gene expression of adenosine-deaminase (ADA), an enzyme that converts ADO into inosine (**Figure 4E**). Specifically, PB from the spleen and LN of mice infected with *T. cruzi*, obtained at 20 Dpi, exhibited a higher expression level of ADA-encoding gene, compared with naive or activated B cells (**Figure 4E**). In turn, activated B cells showed greater expression than naive cells, suggesting a correlation between cellular activation/differentiation and increased ADA gene expression, with levels rising as activation progresses. Moreover, in comparison with naïve B cells, PB also had an increased expression of adenosine-deaminase ADAT2/ADAT3 encoding genes, which form a complex contributing to ADO-to-inosine deamination on tRNA (27), related to antibody production (28) (**Figure 4E**).

### ADO administration in infected animals does not affect ASC response but reduces the GC reaction

**Figure 5:**
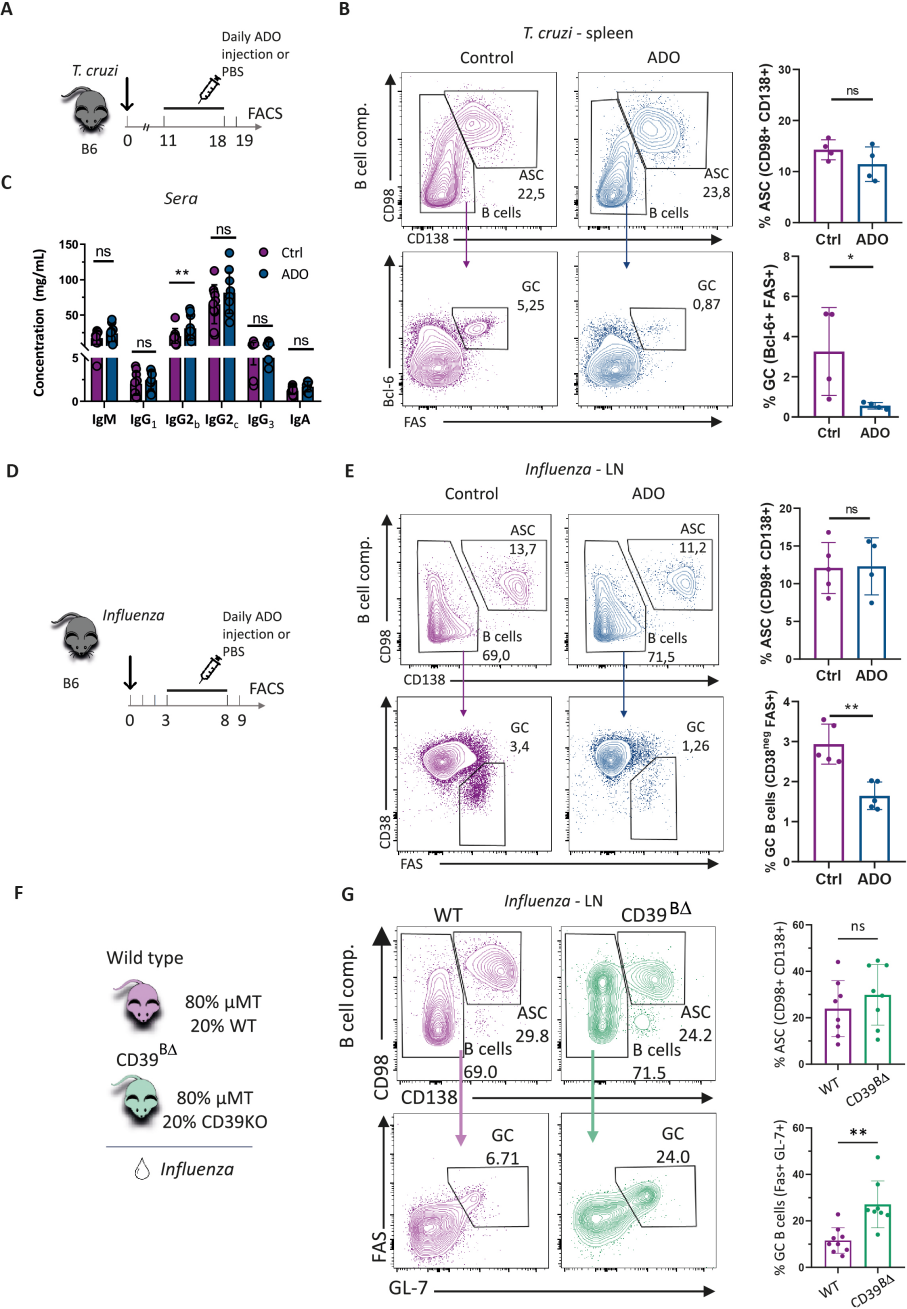
ADO administration and CD39-conditioned GC reaction *in vivo.* ADO treatment scheme in **(A)** *T. cruzi-* or **(D)** *Influenza-*infected mice. Control mice were injected with PBS instead of ADO. **(B, E)** Representative contour plots and statistical analysis showing the frequency of ASC and GC cells in the B-cell compartment (gated in B220^+^ and B220^int^CD138^+^) from spleens of *T. cruzi*-infected mice obtained at 19 Dpi or from LN of *Influenza*-infected mice obtained at 9 Dpi. **(C)** Serum levels of total immunoglobulins from mice described in **(A)**. **(F)** Scheme of chimera design to specifically generate a B-cell compartment deficient in CD39 and controls. **(G)** Representative contour plots and statistical analysis showing the distribution of ASC and GC in the B-cell compartment (B220^+^ and B220^int^CD138^+^) of LN obtained at 9 Dpi from *Influenza*-infected chimeras. **(A-E)** are representative of two independent experiments with n ≥ 4, analyzed with a T-test. *p<0.05, **p<0.01. **(F)** came from three independent experiments, with a total n=8, analyzed with T-test. *p<0.05, **p<0.01.

The expression of ADO receptors and transporters by ASC, activated B cells, and GC B cells suggests that these populations may be responsive to ADO signaling *in vivo*. To investigate this hypothesis, we administered daily intraperitoneal doses of ADO to infected mice. The treatment spanned from 11 to 18 Dpi for mice infected with *T. cruzi* (**Figure 5A**) and from 3 to 8 Dpi for mice infected with *Influenza* (**Figure 5D**). ADO administration in *T. cruzi*-infected mice did not affect the frequency of ASC (PB) but resulted in impaired GC responses, evidenced by a significant decrease in the frequency of GC B cells compared with PBS-treated controls (**Figure 5B**). ADO injection did not affect the levels of immunoglobulins in the serum of *T. cruzi*-infected mice, except for a slight but significant increase in IgG2b (**Figure 5C**). Similarly, *Influenza*-infected mice treated with ADO showed comparable frequencies of ASC and a significant reduction in GC B cells in LN at 9 Dpi, relative to untreated infected control mice (**Figure 5E**).

To evaluate the possible link between the effect of ADO and CD39 expression in the B-cell progeny, we generated chimeric mice in which B cells were unable to express CD39. For this, a mixture of BM cells from µMT and CD39^−/−^ mice (80:20) were transferred into irradiated recipient µMT mice (1 × 10^6^ cells in total). In these chimeric mice, all B cells lacked CD39 expression (CD39^BΔ^ mice), enabling us to compare their response with chimeric mice in which B cells had the potential to express CD39 (**Figure 5F**). In this particular case, both groups of chimeric mice were infected with *Influenza virus*. Infected CD39^BΔ^ mice exhibited similar frequency of ASC (B220^+^CD138^+^CD98^+^), confirming that CD39 is dispensable for ASC generation. However, we observed a significant increased frequency of GC B cells (GL-7^+^Fas^+^CD138^neg^B220^+^) in mediastinal LN from infected chimera mice when compared with infected controls (**Figure 5G**). Altogether, data from *in vivo* ADO supplementation and chimeric mice supported the notion that CD39 expression within the B-cell lineage constrains the GC response, likely through ADO-mediated mechanisms.

### ADO impairs antibody production by plasmablasts *in vitro*

**Figure 6:**
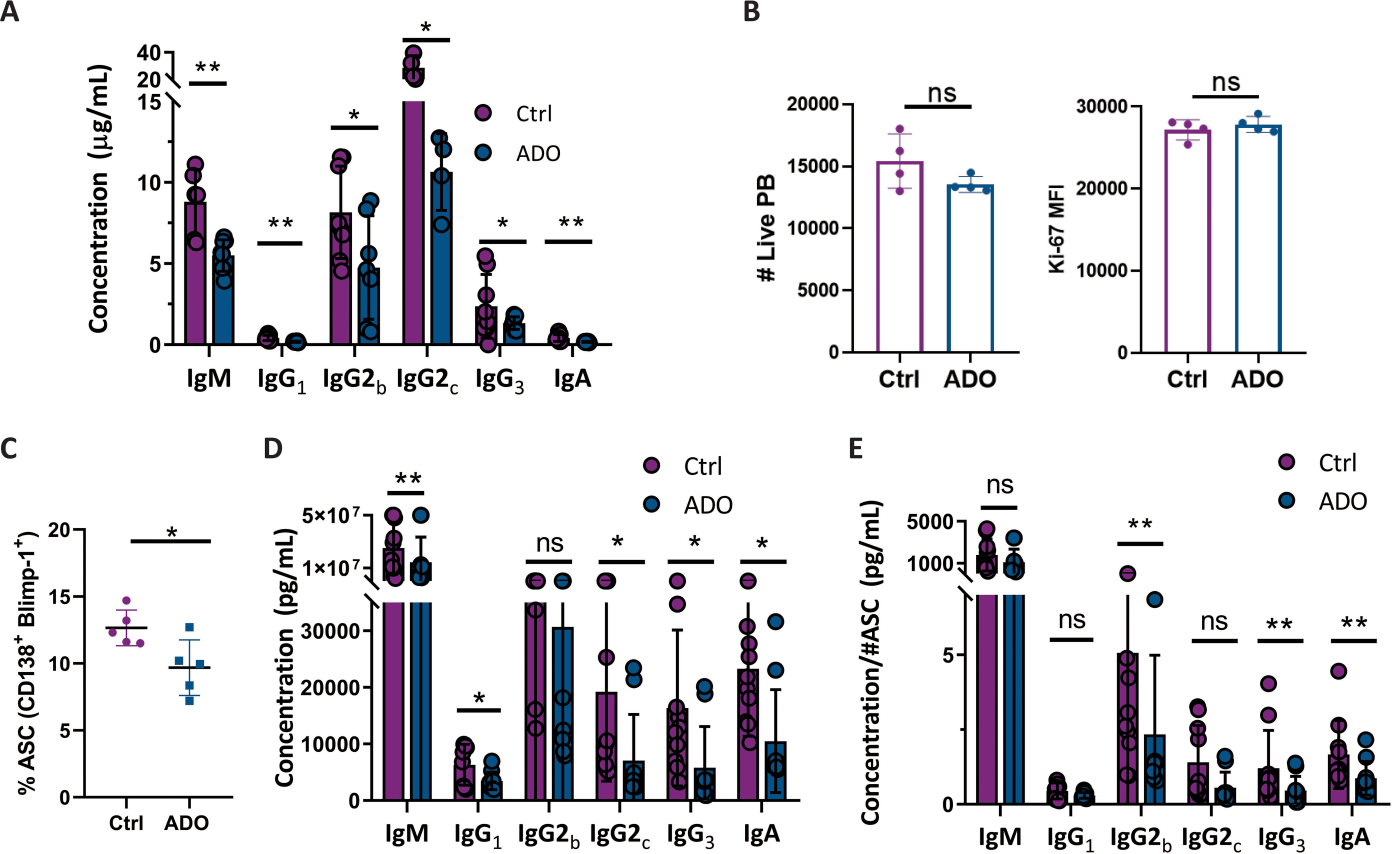
ADO impaired antibody production by PB and by B cells undergoing differentiation *in vivo*. **(A, B)** PB were purified by cell sorting from the spleens of *T. cruzi*-infected mice at 18 Dpi and cultured with ADO (500 µM) or in absence of ADO (Ctrl). **(A)** Total antibody concentration determined by LEGENDplex in the culture supernatant. **(B)** Number of live PB and PB Ki-67 MFI. Data are representative from two independent experiments with n ≥ 4, analyzed with T-test. *p<0.05, **p<0.01. **(C-E)** B cells from non-infected mice were purified by immunomagnetic negative selection and stimulated during 72 h with CpG plus anti-CD40 with ADO (500 µM) or in the absence of ADO (Ctrl). **(C)** Plot with statistical analysis of frequency of live ASC. Total immunoglobulin concentration, determined by LEGENDplex, in culture supernatants of **(D)** total cells or **(E)** normalized according to ASC number. All data are representative from two independent experiments, with n ≥ 5, analyzed with a T-test. *p<0.05, **p<0.01.

To assess the direct effect of ADO on PB, since *in vivo* assays involve the interplay of multiple cell types, we conducted *in vitro* cultures of purified PB in the presence of ADO. For that, sorted splenic PB from *T. cruzi*-infected mice (20 Dpi) were cultured for 24 h in the presence or absence of ADO. ADO exposure resulted in a marked reduction in the concentration of all immunoglobulin isotypes secreted by PB (**Figure 6A**), without affecting their viability or proliferative capacity (**Figure 6B**), as assessed by exclusion of the viability dye and Ki-67 expression, respectively.

### ADO inhibits the activation and differentiation of *in vitro*-activated B cells into ASC

We next examined the impact of ADO on *in vitro*-activated B cells. For that, naïve B cells purified from spleens of non-infected mice were activated *in vitro* using a combination of CpG and anti-CD40, a commonly employed stimulus to promote B-cell activation and differentiation (29, 30), in the presence or absence of ADO. After 72 h of culture, the frequency of generated ASC was analyzed by flow cytometry, and the concentration of Igs in culture supernatants was also determined.

The presence of ADO reduced the frequency of ASC generated in culture (**Figure 6C**), as well as the concentration of most immunoglobulin isotypes in the culture supernatant, with the exception of IgG2b (**Figure 6D**). To determine whether the reduction in immunoglobulin levels was solely due to the decreased ASC or if ADO also directly affected the secretory capacity of the cells, we normalized Ig concentrations to the number of ASC. This analysis revealed that ADO also impaired ASC secretory capacity, as the concentrations of IgG2b, IgG3, and IgA remained reduced after normalization (**Figure 6E**).

## Discussion

In this study, we show that ASC, both under physiological conditions and those generated in response to various infections across different tissues, consistently display high levels of CD39 expression. This high CD39 expression reliably serves as a marker for identifying different types of ASC, including PB and PC. CD39 was detected in ASC irrespective of the immunoglobulin isotype secreted. Importantly, the high expression of CD39 allowed us to identify ASC in the peritoneal cavity of *T. cruzi*-infected mice that cannot be detected by CD138 expression, as this marker is absent in these cells (21).

Our findings extend previous reports by showing that CD39 expression is not limited to ASC in sepsis and autoimmune conditions, as previously described (13, 17), but is also observed in ASC across various infections, anatomical locations, and in both mouse and human models. This expands the scope of CD39 as a marker for identifying ASC in diverse immunological contexts. In other B-cell subsets, such as activated B cells and GC B cells, CD39 expression was virtually undetectable. These results obtained raise new questions about the role of CD39 in ASC and the implication of purinergic signaling in B-cell immunobiology. Regardless of the new questions that have emerged, the general conclusion of our work is that the robust expression of CD39 provides a valuable tool for identifying ASC in conjunction with other markers such as Blimp-1, CD98, TACI, and CD138, among others, particularly in tissues such as in the lung (**Supplementary Figure S2C**), where enzymatic tissue digestions may pose technical challenges by influencing surface expression of certain markers such as CD138.

Despite that high levels of expression of CD39 are known to be present on ASC (13, 17), the exact function of this ectoenzyme in the regulation of ASC and B-cell biology remains unclear. Prior work has described lower levels of xenoreactive IgG antibodies generated in CD39 null mice (24). Other earlier experiments have shown that these mutant mice exhibit impaired B-cell memory responses to T-dependent antigens and increased frequency of somatic mutations post-immunization (25). We observed that the expression of CD39 was not essential for the differentiation of B cells into ASC. The presence of IgA and IgG in CD39^−/−^ mice indicates that this ectoenzyme is not involved in CSR, but it may play a role in regulating antibody secretion, as its absence affects the concentrations of IgG2, IgG3, and IgA produced by splenic PB. Interestingly, in the spleen of infected CD39^−/−^ mice, the frequency of IgM^+^ PB was significantly higher than in WT mice, a difference that was not observed in LN. The differences in the frequency of IgM^+^ PB between tissues may be due to variations in stromal support, local immune cell composition, and routes of antigen delivery (blood vs. lymph), which have been reported to influence the characteristics of immune cell populations (31).

Furthermore, our results revealed that chimeric mice in which B cells did not express CD39 exhibited a comparable frequency of ASC but had significantly elevated frequencies of GC B cells. This indicates that CD39 could play an important role in limiting GC reaction. Our findings demonstrating that CD39 present in ACS is functional suggest that CD39 could exert its effects via the enzymatic breakdown of extracellular nucleotides into ADO through phosphohydrolysis.

In this way, ADO could interact directly with B cells (18), including activated B cells, GC B cells, and PB, as these cells express different ADO receptors. ADO could also impact GC reaction indirectly by inhibiting other mediators involved in the GC response, such as T follicular helper (Tfh) cells. An inverse correlation between Tfh cell generation and the expression of CD39 has also been described (26).

In this study, we demonstrated that the whole ASC populations expressed CD39 whereas a fraction of the ASC also expressed CD73; thus, ASC had the necessary catalytic machinery for the generation of ADO. Moreover, *in vivo*, neighboring cells close to the ASC such as CD4^+^ or CD8^+^ T cells may contribute in a paracrine manner or may provide CD73 functionality for ADO generation (25, 27). We observed that CD39 expressed on PB was biologically active, as WT PB scavenged ATP, more than did CD39^−/−^ PB, suggesting that ASC can generate ADO from extracellular ATP. It is possible that ASC in different tissues may differentially handle the effects of ATP, which is subsequently converted into ADO. This hypothesis is supported by the distribution/composition of CD73^+^ cells in the spleen and LN, as well as by the expression of SLC29A3 and ADA transcripts in ASC from both tissues. Taking into account that PB from *T. cruzi*-infected mice expressed receptors and transporters for ADO, ADO may directly affect ASC by acting as a modulator of their own response/function.

ADO administration in *T. cruzi*-infected mice impaired GC reaction but did not modify the frequency of ASC, probably because they are extrafollicular PB that do not depend on the CG reaction (19). Although PB and GC B cells share some ADO receptors, a clear *in vivo* effect of ADO was observed on the GC response, but not on PB frequency. In this context, GC B cells appear to be more susceptible to the immunomodulatory effects of ADO. This differential sensitivity to ADO of PB and GC B cells is likely attributable to intrinsic features of each population (32) but also to differences in tissue architecture, oxygen availability, cellular conformation, as well as differential expression of the ADORA2a (**Figure 4C**). GC B cells are densely packed, are hypoxic, and undergo apoptosis as part of the selection process, which affects their metabolism and signaling (33). In contrast, extrafollicular PB are more diffusely organized, which likely underlies their differential responsiveness (34). Additionally, it is possible that the effect of ADO, *in vivo*, on the GC reaction is not only direct but also mediated through Tfh cells, as we observed that Tfh cells also express the ADO receptor ADORA2a (data not shown).

With respect to ADO receptors, ADORA2a functions as an immunological “brake” triggered by ADO, modulating inflammation and cellular responses. Its expression on a given cell makes the cell susceptible to regulation. In contrast, ADORA2b, which has lower affinity for ADO, is activated under tissue stress or hypoxia and promotes the release of IL-6 and VEGF. ADORA3 can have pro- or anti-inflammatory effects depending on the context and can induce apoptosis in certain tumor cells (reviewed in (35). Based on this information, the effect of ADO on ASC may result from the combined interaction of ADO with the different receptors. It is possible that more than one receptor is activated simultaneously, so that the observed outcome depends not only on this joint activation but also on the concentration of ADO in the medium.

Regarding the effect of ADO on ASC (PB) purified from the spleens of infected mice and on ASC generated *in vitro* from activated B cells, we observed that ADO impaired the secretory function of ASC while preserving their survival. *In vitro*, ADO also reduced the frequency of ASC derived from activated cells. In contrast to the findings *in vivo*, where ADO treatment did not affect immunoglobulin levels, *in vitro* approaches demonstrated that ADO is able to inhibit immunoglobulin production when acting directly on PB. It is likely that higher doses of ADO are required to inhibit antibody secretion *in vivo*, which could occur in infections or pathological conditions where high levels of ATP, released from dying or stressed cells, are available (36, 37).

It is possible that CD39 may have specific additional functions depending on the type of ASC. Thus, its expression in extrafollicular PB may confer a novel regulatory property beyond those already described through PD-L1 expression, which affects T-cell response (19), and nutrient consumption that impairs the GC reaction (28). In long-lived ASC, it is possible that CD39 contributes to their persistence in the BM, potentially by modulating local purinergic signaling, which is known to support plasma cell survival (38).

In summary, our findings suggest that elevated CD39 expression characterizes ASC and contributes to ADO production, which may serve as an intrinsic regulatory mechanism controlling antibody secretion and the GC response. These novel data highlight the relevance of the purinergic pathway in antibody-mediated immune responses and uncover a novel regulatory axis in B-cell biology.

## Materials and methods

### Mice

C57BL/6 mice used for experiments performed in Argentina were initially obtained from The Jackson Laboratories (USA) and C57BL/6, and uMT mice used for experiments performed in Marseille (France) were obtained from Janvier Labs (France). CD39^−/−^ mice on C57BL/6 background were provided by Dr Simon Robson (Transplant Institute and Hepatology, Department of Medicine, Beth Israel Deaconess Medical Center and Harvard Medical School, Boston, USA). These mice were housed and bred in the Animal Facility of the CIBICI-CONICET, FCQ-UNC for at least 6 years.

Mice used in experiments conducted at the John Curtin School of Medical Research (JCSMR) were as follows.

SW_HEL_ CD45.1 mice which carry a Vk10k light-chain transgene and a knocked-in VH10 Ig heavy chain in place of the JH segments of the endogenous IgH gene that encode a high-affinity antibody for HEL were obtained from the laboratory of R. Brink (Garvan Institute, Sydney, New South Wales, Australia).

MRL/*Faslpr/lpr* (stock number: 000485) are homozygous for the lymphoproliferation spontaneous mutation (*Faslpr*) and are a model to study systemic lupus erythematosus (SLE) and Sjogren (Sicca) syndrome.

*Roquin*san/san C57BL/6 mice: The Sanroque mouse results from homozygous missense mutations in a ubiquitin ligase family member, roquin (Rc3h1), which produces a florid lupus phenotype.

Mouse experiments conducted at the CIBICI were approved by and performed by the guidelines of the Institutional Animal Care and Use Committee of the FCQ-UNC (Approval Number HCD RD-2021-2033-E-UNC-DEC#FCQ). Mouse experiments conducted at JCSMR were approved by the Animal Experimentation Ethics Committee (Australian National University protocol number 2016/17). Experiments performed in CIML (Marseille, France) were conducted by French and European guidelines for Animal Care under permission number 16708–2018091116493528 following review and approval by the local animal ethics committee in Marseille.

Male and female mice were used at age-matched (8–12 weeks old) and housed with a 12-h light–dark cycle. Mice from all institutions were maintained in specific pathogen-free conditions and had access to food and water ad libitum. The animals in each experiment were housed in cages located on the same racks and at a similar height, and, when possible, animals from different groups were mixed in the same cages to avoid nuisance variables. Animal care staff were unaware of allocation groups. Prior to sample collection, a person not involved in the process assigned codes to each animal to ensure blind handling during both the experiments and the analysis.

In all cases, animals that showed poor health, suffering, or excessive weight loss were euthanized.

### Pathogens and experimental infections

*T. cruzi* infection: Mice were intraperitoneally (ip) inoculated with 5×10^3^ trypomastigotes of *T. cruzi* (Tulahuén strain)/0.2 ml PBS (39). Uninfected littermates were injected with PBS and processed in parallel. Spleen and LN were collected on different days post-infection (Dpi) for immune response analysis. Bone marrow (BM) cells of *T. cruzi*-infected mice were isolated by flushing femurs and tibias of mice with RPMI-1640.

*Influenza* infection: Mice were anesthetized ip with ketamine/xylazine (100 mg/kg body) and intranasally infected with 5 PFU of *Influenza virus* A/Puerto Rico/8/1934 (PR8) H1N1 strain (22). Mediastinal LN were obtained after 9 Dpi, and LN, spleen, and BM were obtained at 3 months pi with *Influenza* virus to evaluate ASC response. In reinfection experiments, mice were rechallenged with 5.10^4^ PFU of influenza H3N2 X31 strain and evaluated at 4 Dpi.

*Staphylococcus aureus* infection: Mice were intradermally inoculated in the ear with 10^7^ CFU of *S. aureus* (ATCC BAA-1556) in 5 μl of PBS, and draining LN from 10 Dpi *S. aureus-*infected mice was obtained (40).

### Bone marrow chimeras

For the generation of mixed bone marrow chimeras, μMT mice of 6–8 weeks of age were irradiated with two doses of 4.75 Gy, 4 h apart. One day later, a mixture of BM cells from µMT and WT mice (80:20) or BM cells from µMT mice plus CD39^−/−^ (80:20) was administered through i.v. injection into irradiated recipient µMT mice (1×10^6^ cells in total). Experimental animals were kept in the water with Bactrim for 3 days prior and 3 weeks post-irradiation treatment. Chimeras were infected with *influenza virus* after 8 weeks of reconstitution and the frequency of cells from the B-cell compartment was evaluated by FACS at 9 Dpi (41).

### Immunizations

To evaluate the CD39 expression on ASC generated by TD-antigen immunization, C57BL/6 mice were transferred, by intravenous (iv) injection, with splenocytes from CD45.1 SWHel mice (Het/Het) containing 30.000 CD19^+^Hel^+^ cells simultaneously with 2×10^8^ Hel-conjugated SRBCs (Applied Biological Products Management, Australia). HEL was conjugated with AlexaFluor647 with a protein labeling kit (Invitrogen). Frequency and phenotype of ASC were analyzed at 7 days post-immunization.

### *In vivo* ADO treatment

ADO ≥99% (Sigma-Aldrich, A9251) was used. Daily intraperitoneal doses of ADO were administered to infected mice. The treatment ranged from 11 to 18 Dpi for 10 mice infected with *T. cruzi* and from 3 to 8 Dpi for those infected with *Influenza*. Mice were weighed before each administration, and the daily dose was 25 mg/kg, diluted in PBS. Control animals were injected with daily doses of PBS. The injection site was alternated to minimize damage.

### COVID-19 patients

Before their participation, all enrolled patients provided written informed consent by the Declaration of Helsinki, and their data were protected as per Argentine law N° 25.326. Patient recruitment took place during the first and second waves of the SARS-CoV-2 pandemic in Córdoba, Argentina, between October-December 2020 and February-June 2021, respectively. Diagnosis of SARS-CoV-2 infection was performed using a nucleic acid amplification test for SARS-CoV-2, by the guidelines provided by the Argentine Health Ministry. Reverse transcriptase polymerase chain reaction (RT-PCR) (PerkinElmer^®^, Massachusetts, U.S.) analysis was conducted on samples obtained from nasal and pharyngeal swabs. Only hospitalized, primo-infected unvaccinated patients were recruited.

Peripheral blood from healthy donors (HD) and COVID-19 patients on hospitalization was drawn by venipuncture into BD Vacutainer^®^ EDTA tubes BD. Peripheral blood mononuclear cells (PBMCs) were isolated from whole blood samples using Ficoll-Paque™ PLUS (GE Healthcare Bio-Sciences AB). Isolated PBMCs were cryopreserved in heat-inactivated fetal bovine serum (FBS; Natocor) containing 10% DMSO (Sigma-Aldrich) and stored in liquid nitrogen until use.

### Cell preparation

Spleens, LN, or BM from infected or immunized mice were obtained, and tissues were homogenized through a 0.70-µm cell strainer. Total peritoneal cells were obtained from washes of the peritoneal cavity with 2% FBS-PBS. Erythrocytes in cell suspensions were lysed for 5 min in Tris–ammonium chloride buffer. Viable cell numbers were determined by trypan blue exclusion using a hemocytometer.

### Flow cytometry

For surface staining, single-cell suspensions were washed first in ice-cold PBS and incubated for 10 min with live/dead staining. Cells were washed in PBS and then incubated with fluorochrome-labeled Abs for 30 min at 4 °C (surface staining). For intracellular staining, cells were fixed and permeabilized with BD Cytofix/Cytoperm and Perm/Wash (BD Biosciences) according to the manufacturer’s instructions. Data were collected on a BD FACSCanto II, BD LSR II, and BD Fortessa X20 and were analyzed using the FlowJo software (TreeStar). The specific cell populations and gating strategy in each case are described in different **Supplementary Figures** (appropriately mentioned in the corresponding Result section).

### Antibodies

The following anti-mouse antibodies were used for FACS: CD19-PE (6D5), CD19-BV605 (6D5), CD3-AlexaFluor700 (17A2), CD3-APCCY7 (17A2), CD4-APC-Cy7 (GK1.5), CD8-PerCP-Cy5.5 (53-6.7), CD8-FITC (5H10-1), Streptavidin BV605, PD-1-BV421 (29F.1A12), PDL-1-Pecy7 (10F.9G2), Bcl6-PE-Dazzle594 (7D1), ICOS-AlexaFluor488 (C398.4A), CD45.1- BV605 (A20), B220-APCCy7 (RA3-6B2), CD11b-PE-Cy7 (M1/70), CD5-PE (53-7.3), and Live Dead Aqua 430 were from BioLegend; CD19-AlexaFluor700 (1D3), CD19-FITC (1D3), IgD-FITC (11-26c), CD8-PECy7 (53-6.7), TNF-APC (MP6-XT22), and PD-L1-PE (MIH5) were from eBioscience; and CD138-BV605 (281-2), CD138-APC (281-2), CD138-PE (281-2), CD138-Biotin, CD38-BV421 (90/CD38), CXCR5-Biotin (2G8), IFN-γ-FITC (XMG1.2), Ki-67-AlexaFluor647 (B56), Blimp-1-PE-CF594 (5E7), and CD8-BUV805 (53-6.7) were from BD Biosciences.

The following anti-mouse antibodies were used for tissue immunofluorescence: B220-PE (RA3-6B2), FITC anti-CD3 (145-2C11) and antiCD39-APC (143809) from BioLegend and CD138-APC (281-2) from BD Biosciences.

The following anti-human antibodies were used for FACS: CD39-SB436 (eBioA1) from eBioscience; CD45-APC-Cy7 (2D1), CD19-BV605 (HIB19), and CD3-BV650 (UCHT1) from BioLegend; and CD24-FITC (ML5) and CD38-APC (HIT2) from BD Biosciences.

### Immunofluorescence

For tissue immunofluorescence, mediastinal LN from *Influenza virus*-infected WT mice were fixed in 4% PFA for 6 h at 4°C, washed with PBS, incubated overnight in PBS 30% sucrose solution, immersed in OCT, and snap-frozen in liquid nitrogen-cooled isopentane. Cryostat sections (10 mm thick) were dried in silica beads, permeabilized with PBS Saponin 0.5% for 30 min, and blocked with PBS 0.5% saponin 2% BSA 1% goat serum 1% FCS for 30 min. Sections were then incubated with antibodies in PBS 0.5% saponin 2% BSA 1% goat serum 1% FCS for at least 1 h, washed, and mounted in Fluoromount-G mounting media. Imaging was carried out on a LSM 780 (Zeiss) inverted confocal microscope using a Plan-Apochromat 20×/0.8 M27 objective (22).

### Cell sorting

Spleen and inguinal LN from WT or CD39^−/−^ *T. cruzi*-infected mice were obtained at 18 or 20 Dpi, and different cell populations were purified by cell sorting in an Aria II BD cytometer. ASC as B220^int^CD138^+^ CD98^+^, naive B cells as B220^+^IgD^+^, and activated B cells as B220^+^IgD^neg^CD138^neg^ were sorted from spleens or LN (gated on live lymphocytes).

### RNA sequencing

ASC (specifically PB), naive B cells, and activated B cells from *T cruzi*-infected mice were obtained at 20 Dpi from inguinal LN and spleen by cell sorting as we described before, and total RNA was extracted with the Arcturus^®^ PicoPure^®^ RNA Isolation Kit, as indicated by the manufacturer in its User Guide. RNA sequencing (RNA-Seq) was performed by RNA-Seq services by GENEWIZ Multiomics & Synthesis Solutions from Azenta Life Sciences (NJ, USA). PolyA selection for mRNA species was used for rRNA removal and GENEWIZ performed an ultra-low input library preparation.

For the analysis, sequence reads were trimmed to remove possible adapter sequences and nucleotides with poor quality using Trimmomatic v.0.36. The trimmed reads were mapped to the Mus musculus GRCm38 reference genome available on ENSEMBL using the STAR aligner v.2.5.2b. Using DESeq2, a comparison of gene expression between the customer-defined groups of samples was performed. The Wald test was used to generate p-values and log2 fold changes. Genes with an adjusted p-value < 0.05 and absolute log2 fold change >1 were called differentially expressed genes for each comparison.

### Cell culture

1 × 10^5^ purified PB (ASC) were cultured overnight with complete media plus BAFF (100 ng/mL) with or without ADO ≥99% (Sigma-Aldrich, A9251) at a final concentration of 500 µM (42) and then stimulated with PMA-ionomycin for 5 h. Culture supernatants were collected for antibody determination.

Splenic B cells from non-infected mice were isolated using a negative selection kit (StemCell, Cat#19854) according to the manufacturer’s instructions. A total of 2×10^5^ B cells were cultured in complete RPMI with 2 μg/mL CpG (Eurofins Genomics) and 2 μg/mL anti-CD40 (BioLegend) for 24 h at 37 °C in a 96-well plate, in the presence or absence of ADO ≥99% (Sigma-Aldrich, A9251), at a final concentration of 500 µM. After stimulation, cells were washed, counted, and evaluated by flow cytometry.

### Immunoglobulin concentrations

Immunoglobulins (IgM, IgG_1_, IgG_2b_, IgG_2c_, IgG_3_, and IgA) were quantified using a bead-base multiplex assay by FACS (LEGENDplex™, Mouse Immunoglobulin Isotyping Panel, 6 plex) in the culture supernatant of WT and CD39^−/−^ PB. PB supernatants and standards were run in duplicates in plates according to the manufacturer’s instructions (BioLegend). The samples were transferred from plate to tube and read using a FACSCanto II cytometer and data were analyzed with LEGENDplex™ Data Analysis Software.

### Determination of *T. cruzi* specific Abs

*T. cruzi*-specific Abs were quantified by ELISA using *T. cruzi* lysate-sensitized plates from Wiener lab (Rosario, Argentina). The wells were incubated with supernatants from WT and CD39^−/−^ PB for 2 h at room temperature (RT). Peroxidase-conjugated anti-mouse IgM or IgG isotypes were added and incubated for 1:30 h at RT. The reaction was developed with TMB Substrate Reagent (BD Biosciences) and read in a Microplate Reader 450 from Bio-Rad.

The ELISA test was considered positive if the mean OD value was two standard deviations above the mean value for the control supernatant assayed in parallel.

### ELISpot assay

To measure the frequency of ASC, ELISpot multiscreen filtration plates (Millipore) were activated with absolute ethanol, washed with PBS, and coated overnight at 4°C with 10 μg/mL of the isotype-specific goat anti-mouse IgM, IgG, and IgA (Southern Biotech), for total ASC detection, overnight at 4°C and blocked with 1% BSA. Plates were subsequently blocked for 2 h with complete medium and incubated for 24 h at 37 °C with serial dilutions of splenic single-cell suspensions. Plates were washed with PBS 0.01% Tween-20 and incubated for 1 h with 100 µL of 1 µg/mL of biotin-conjugated anti-IgM, IgG, or IgA (SouthernBiotech) diluted in PBS 1% BSA. Then, plates were washed with 0.1% Tween 20 and incubated with 100 µL of 1 µg/mL ExtrAvidin-alkaline phosphatase (Sigma-Aldrich) in PBS 1% BSA. Spots were detected using BCIP/NBT (Sigma-Aldrich).

### CD39 function *in vivo*: extracellular ATP hydrolysis assay

To test whether CD39 on PB cells is a functional E-NTPDase, purified PB from the spleen of *T. cruzi*-infected mice was incubated with 10 μM ATP in culture medium without serum, and the changes in extracellular ATP concentrations, after 40 min at 37°C and 5% CO_2_, was measured with an ATP bioluminescent assay kit (Invitrogen A22066) following the manufacturer’s instructions. A cell-free medium with ATP alone was used as a control. The values obtained from each experimental condition will be referred to as the value from the medium culture alone with ATP, which is considered 100% of ATP.

### Statistics

Statistical significance through comparison of mean values was assessed by a two-tailed Student’s t-test, one-way ANOVA, or two-way ANOVA followed by Bonferroni’s posttest using GraphPad software.

## Data Availability

The original contributions presented in the study are publicly available in the NCBI repository. These data can be found here: https://www.ncbi.nlm.nih.gov/bioproject/1345747.

## References

[1] Tellier J Nutt SL. Plasma cells: The programming of an antibody-secreting machine. Eur J Immunol. (2019) 49:30–7. doi: 10.1002/eji.201847517, PMID: 30273443

[2] Bermejo DA Jackson SW Gorosito-Serran M Acosta-Rodriguez EV Amezcua-Vesely MC Sather BD. Trypanosoma cruzi trans-sialidase initiates a program independent of the transcription factors RORγt and Ahr that leads to IL-17 production by activated B cells. Nat Immunol. (2013) 14:514–22. doi: 10.1038/ni.2569, PMID: 23563688 PMC3631452

[3] Pelletier N McHeyzer-Williams LJ Wong KA Urich E Fazilleau N McHeyzer-Williams MG. Plasma cells negatively regulate the follicular helper T cell program. Nat Immunol. (2010) 11:1110–8. doi: 10.1038/ni.1954, PMID: 21037578 PMC3058870

[4] Fillatreau S. Natural regulatory plasma cells. Curr Opin Immunol. (2018) 55:62–6. doi: 10.1016/j.coi.2018.09.012, PMID: 30292126 PMC6290076

[5] Giovannini D Belbezier A Baillet A Bouillet L Kawano M Dumestre-Perard C. Heterogeneity of antibody-secreting cells infiltrating autoimmune tissues. Front Immunol. (2023) 14:1111366. doi: 10.3389/fimmu.2023.1111366, PMID: 36895558 PMC9989216

[6] Granell M Calvo X Garcia-Guiñón A Escoda L Abella E Martínez CM. Prognostic impact of circulating plasma cells in patients with multiple myeloma: implications for plasma cell leukemia definition. Haematologica. (2017) 102:1099–104. doi: 10.3324/haematol.2016.158303, PMID: 28255016 PMC5451342

[7] McGettigan SE Debes GF. Immunoregulation by antibody secreting cells in inflammation, infection, and cancer. Immunol Rev. (2021) 303:103–18. doi: 10.1111/imr.12991, PMID: 34145601 PMC8387433

[8] Shlomchik MJ Weisel F. Germinal center selection and the development of memory B and plasma cells. Immunol Rev. (2012) 247:52–63. doi: 10.1111/j.1600-065X.2012.01124.x, PMID: 22500831

[9] Tarlinton DM Ding Z Tellier J Nutt SL. Making sense of plasma cell heterogeneity. Curr Opin Immunol. (2023) 81:102297. doi: 10.1016/j.coi.2023.102297, PMID: 36889029

[10] Kallies A Hasbold J Tarlinton DM Dietrich W Corcoran LM Hodgkin PD. Plasma cell ontogeny defined by quantitative changes in blimp-1 expression. J Exp Med. (2004) 200:967–77. doi: 10.1084/jem.20040973, PMID: 15492122 PMC2211847

[11] Pracht K Meinzinger J Daum P Schulz SR Reimer D Hauke M. A new staining protocol for detection of murine antibody-secreting plasma cell subsets by flow cytometry. Eur J Immunol. (2017) 47:1389–92. doi: 10.1002/eji.201747019, PMID: 28608550

[12] Tellier J Nutt SL. Standing out from the crowd: How to identify plasma cells. Eur J Immunol. (2017) 47:1276–9. doi: 10.1002/eji.201747168, PMID: 28787106

[13] Dang VD Mohr E Szelinski F Le TA Ritter J Hinnenthal T. CD39 and CD326 are bona fide markers of murine and human plasma cells and identify a bone marrow specific plasma cell subpopulation in lupus. Front Immunol. (2022) 13:873217. doi: 10.3389/fimmu.2022.873217, PMID: 35464469 PMC9024045

[14] Antonioli L Pacher P Vizi ES Haskó G. CD39 and CD73 in immunity and inflammation. Trends Mol Med. (2013) 19:355–67. doi: 10.1016/j.molmed.2013.03.005, PMID: 23601906 PMC3674206

[15] Zacca ER Amezcua Vesely MC Ferrero PV Acosta CDV Ponce NE Bossio SN. B cells from Patients with Rheumatoid Arthritis Show Conserved CD39-Mediated Regulatory Function and increased CD39 Expression After Positive Response to Therapy. J Mol Biol. (2021) 433:166687. doi: 10.1016/j.jmb.2020.10.021, PMID: 33098857 PMC9376888

[16] Veh J Ludwig C Schrezenmeier H Jahrsdörfer B. Regulatory B cells—Immunopathological and prognostic potential in humans. Cells. (2024) 13:357. doi: 10.3390/cells13040357, PMID: 38391970 PMC10886933

[17] Nascimento DC Viacava PR Ferreira RG Damaceno MA Piñeros AR Melo PH. Sepsis expands a CD39+ plasmablast population that promotes immunosuppression via adenosine-mediated inhibition of macrophage antimicrobial activity. Immunity. (2021) 54:2024–41. doi: 10.1016/j.immuni.2021.08.005, PMID: 34473957

[18] Schena F Volpi S Faliti CE Penco F Santi S Proietti M. Dependence of immunoglobulin class switch recombination in B cells on vesicular release of ATP and CD73 ectonucleotidase activity. Cell Rep. (2013) 3:1824–31. doi: 10.1016/j.celrep.2013.05.022, PMID: 23770243

[19] Serrán MG Vernengo FF Almada L Beccaria CG Gazzoni Y Canete PF. Extrafollicular plasmablasts present in the acute phase of infections express high levels of PD-L1 and are able to limit T cell response. Front Immunol. (2022) 13:828734. doi: 10.3389/fimmu.2022.828734, PMID: 35651611 PMC9149371

[20] Kaku H Cheng KF Al-Abed Y Rothstein TL. A novel mechanism of B cell–mediated immune suppression through CD73 expression and adenosine production. J Immunol. (2014) 193:5904–13. doi: 10.4049/jimmunol.1400336, PMID: 25392527 PMC4321875

[21] Merino MC Montes CL Acosta-Rodriguez EV Bermejo DA Amezcua-Vesely MC Gruppi A. Peritoneum from Trypanosoma cruzi-infected mice is a homing site of Syndecan-1 neg plasma cells which mainly provide non-parasite-specific antibodies. Int Immunol. (2010) 22:399–410. doi: 10.1093/intimm/dxq019, PMID: 20207717

[22] Gregoire C Spinelli L Villazala-Merino S Gil L Holgado MP Moussa M. Viral infection engenders bona fide and bystander subsets of lung-resident memory B cells through a permissive mechanism. Immunity. (2022) 55:1216–1233.e9. doi: 10.1016/j.immuni.2022.06.002, PMID: 35768001 PMC9396418

[23] Wu Y Sun X Kaczmarek E Dwyer KM Bianchi E Usheva A. RanBPM associates with CD39 and modulates ecto-nucleotidase activity. Biochem J. (2006) 396:23–30. doi: 10.1042/BJ20051568, PMID: 16478441 PMC1449986

[24] Robson SC Enjyoji K Goepfert C Imai M Kaczmarek E Lin Y. Modulation of extracellular nucleotide-mediated signaling by CD39/nucleoside triphosphate diphosphohydrolase-1. Drug Dev Res. (2001) 53:193–207. doi: 10.1002/ddr.1188

[25] Dwyer KM Deaglio S Gao W Friedman D Strom TB Robson SC. CD39 and control of cellular immune responses. Purinergic Signal. (2007) 3:171. doi: 10.1007/s11302-006-9050-y, PMID: 18404431 PMC2096766

[26] Cao W Fang F Gould T Li X Kim C Gustafson C. Ecto-NTPDase CD39 is a negative checkpoint that inhibits follicular helper cell generation. J Clin Invest. (2020) 130:3422–36. doi: 10.1172/JCI132417, PMID: 32452837 PMC7324201

[27] Deng W Li Y Ma S Mao L Yu G Bu L. Specific blockade CD 73 alters the “exhausted” phenotype of T cells in head and neck squamous cell carcinoma. Intl J Cancer. (2018) 143:1494–504. doi: 10.1002/ijc.31534, PMID: 29663369 PMC11523565

[28] Vijay R Guthmiller JJ Sturtz AJ Surette FA Rogers KJ Sompallae RR. Infection-induced plasmablasts are a nutrient sink that impairs humoral immunity to malaria. Nat Immunol. (2020) 21:790–801. doi: 10.1038/s41590-020-0678-5, PMID: 32424361 PMC7316608

[29] Izadi N Hauk PJ. Cellular assays to evaluate B-cell function. J Immunol Methods. (2023) 512:113395. doi: 10.1016/j.jim.2022.113395, PMID: 36470409

[30] Heath AW Wu WW Howard MC. Monoclonal antibodies to murine CD40 define two distinct functional epitopes. Eur J Immunol. (1994) 24:1828–34. doi: 10.1002/eji.1830240816, PMID: 7519997

[31] Krausgruber T Fortelny N Fife-Gernedl V Senekowitsch M Schuster LC Lercher A. Structural cells are key regulators of organ-specific immune responses. Nature. (2020) 583:296–302. doi: 10.1038/s41586-020-2424-4, PMID: 32612232 PMC7610345

[32] Boothby M Rickert RC. Metabolic regulation of the immune humoral response. Immunity. (2017) 46:743–55. doi: 10.1016/j.immuni.2017.04.009, PMID: 28514675 PMC5640164

[33] Cho SH Raybuck AL Stengel K Wei M Beck TC Volanakis E. Germinal centre hypoxia and regulation of antibody qualities by a hypoxia response system. Nature. (2016) 537:234–8. doi: 10.1038/nature19334, PMID: 27501247 PMC5161594

[34] Elsner RA Shlomchik MJ. Germinal center and extrafollicular B cell responses in vaccination, immunity, and autoimmunity. Immunity. (2020) 53:1136–50. doi: 10.1016/j.immuni.2020.11.006, PMID: 33326765 PMC7748291

[35] Zhang T Yu-Jing L Ma T. The immunomodulatory function of adenosine in sepsis. Front Immunol. (2022) 13:936547. doi: 10.3389/fimmu.2022.936547, PMID: 35958599 PMC9357910

[36] Khakh BS Alan North R. P2X receptors as cell-surface ATP sensors in health and disease. Nature. (2006) 442:527–32. doi: 10.1038/nature04886, PMID: 16885977

[37] Idzko M Ferrari D Eltzschig HK. Nucleotide signalling during inflammation. Nature. (2014) 509:310–7. doi: 10.1038/nature13085, PMID: 24828189 PMC4222675

[38] Conter LJ Song E Shlomchik MJ Tomayko MM. CD73 expression is dynamically regulated in the germinal center and bone marrow plasma cells are diminished in its absence. PloS One. (2014) 9:e92009. doi: 10.1371/journal.pone.0092009, PMID: 24664100 PMC3963874

[39] Fiocca Vernengo F Beccaria CG Araujo Furlan CL Tosello Boari J Almada L Gorosito Serrán M. CD8 ^+^ T cell immunity is compromised by anti-CD20 treatment and rescued by interleukin-17A. mBio. (2020) 11:e00447–20. doi: 10.1128/mBio.00447-20, PMID: 32398312 PMC7218282

[40] Gaya M Barral P Burbage M Aggarwal S Montaner B Warren Navia A. Initiation of antiviral B cell immunity relies on innate signals from spatially positioned NKT cells. Cell. (2018) 172:517–33. doi: 10.1016/j.cell.2017.11.036, PMID: 29249358 PMC5786505

[41] Paus D Phan TG Chan TD Gardam S Basten A Brink R. Antigen recognition strength regulates the choice between extrafollicular plasma cell and germinal center B cell differentiation. J Exp Med. (2006) 203:1081–91. doi: 10.1084/jem.20060087, PMID: 16606676 PMC2118299

[42] Bergero G Mazzocco YL Del Rosso S Liu R Cejas Gallardo ZM Robson SC. Purinergic signaling modulates CD4+ T cells with cytotoxic potential during Trypanosoma cruzi infection. J Clin Invest. (2025) 135:e186785. doi: 10.1172/JCI186785, PMID: 40590226 PMC12208558

