# Supplementary material for: CD39 is an antibody-secreting B-cell marker that modulates germinal center and antibody responses during infection
Source: Front Immunol. 2025 Oct 20;16:1547929. doi: 10.3389/fimmu.2025.1547929 (PMC12580622; doi:10.3389/fimmu.2025.1547929)
Supplement: Supplementary file 1 [file DataSheet1.pdf]

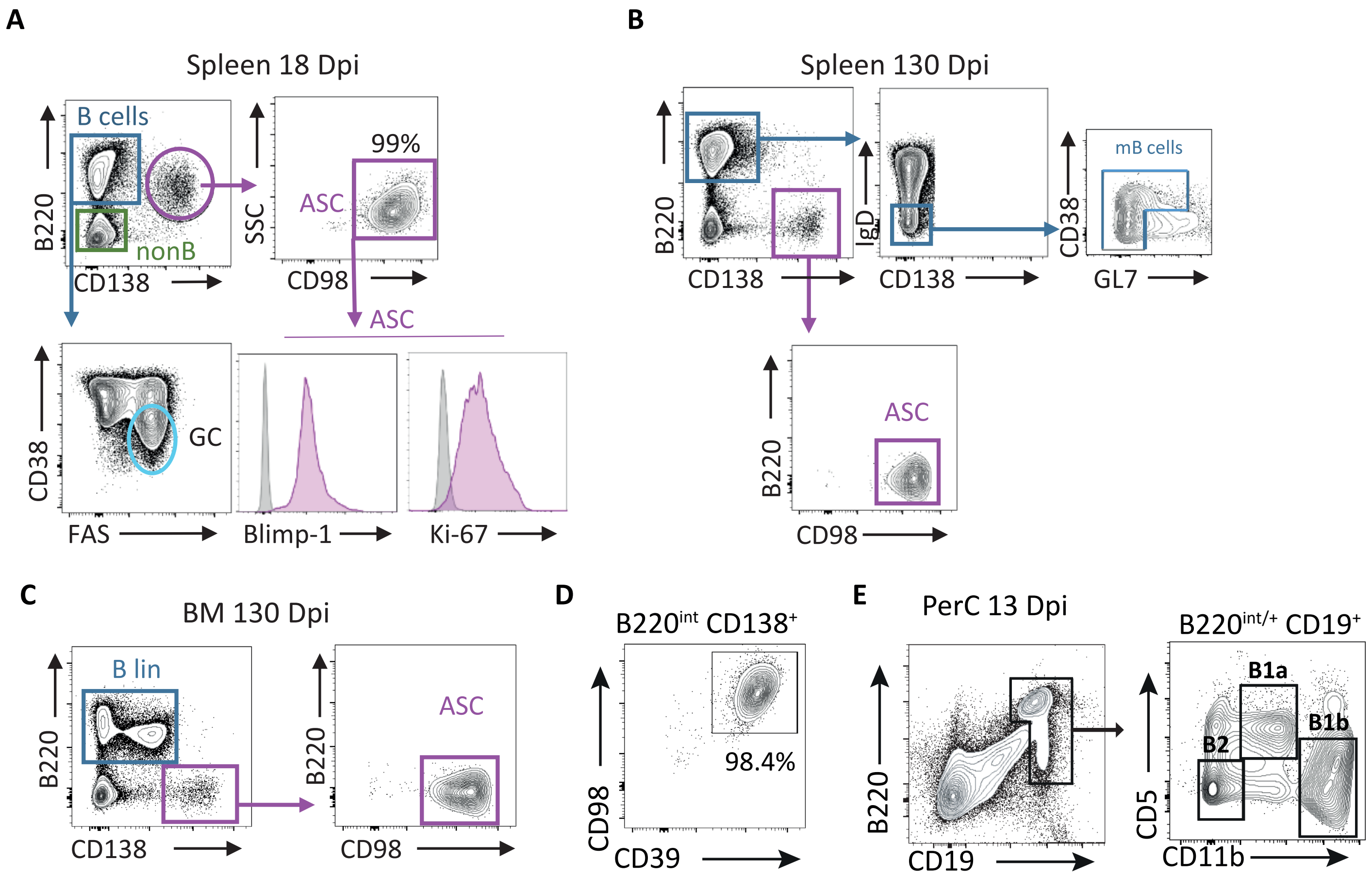

Fig. S1

Gating strategies for the analysis of B cell subsets and ASC in *T. cruzi* infected mice. (A) Flow cytometry gating strategy to identify ASC (CD138^{+}CD98^{+}B220^{int}) and GC B cells (CD138^{neg}CD38^{neg}Fas^{+}B220^{+}) in the spleen of 18 Dpi-*T. cruzi* infected mice. The same gate strategy was used for LN obtained at 24 Dpi. **(B)** Flow cytometry gating strategy to identify mB cells (CD138^{neg}IgD^{neg}B220^{+} excluding CD38^{neg}GL7^{+}) and ASC (CD138^{+}CD98^{+} B220$^{int/neg}$) in the spleen of 130 Dpi-*T. cruzi* infected mice. **(C)** Gating strategy to identify B lin (CD138^{neg} B220^{+}) cells and ASC (CD138^{+}CD98^{+}B220^{neg}) in BM of 130 Dpi-*T. cruzi* infected mice.These gate strategies were used for the analysis shown in Fig.1.
nonB: non-B cells; mB cells: memory B cells; B lin (B cell lineage): B cells and precursors (excluding plasma cells B220^{neg}CD138^{+}). **(D)** Expression of CD39 and CD98 in splenic ASC from 18 Dpi-*T. cruzi* infected mouse determined by flow cytometry. **(E)** Gating strategy to identify B1a (CD5^{+} CD11b^{lo}), B1b (CD5^{neg} CD11b^{+}) and B2 (CD5^{neg} CD11b^{neg}) cells within B220$^{int/+}$ CD19^{+} cells in peritoneal cavity (PerC) of *T. cruzi* infected mice obtained at 15 Dpi.
All gates came from singlets, live lymphocytes.

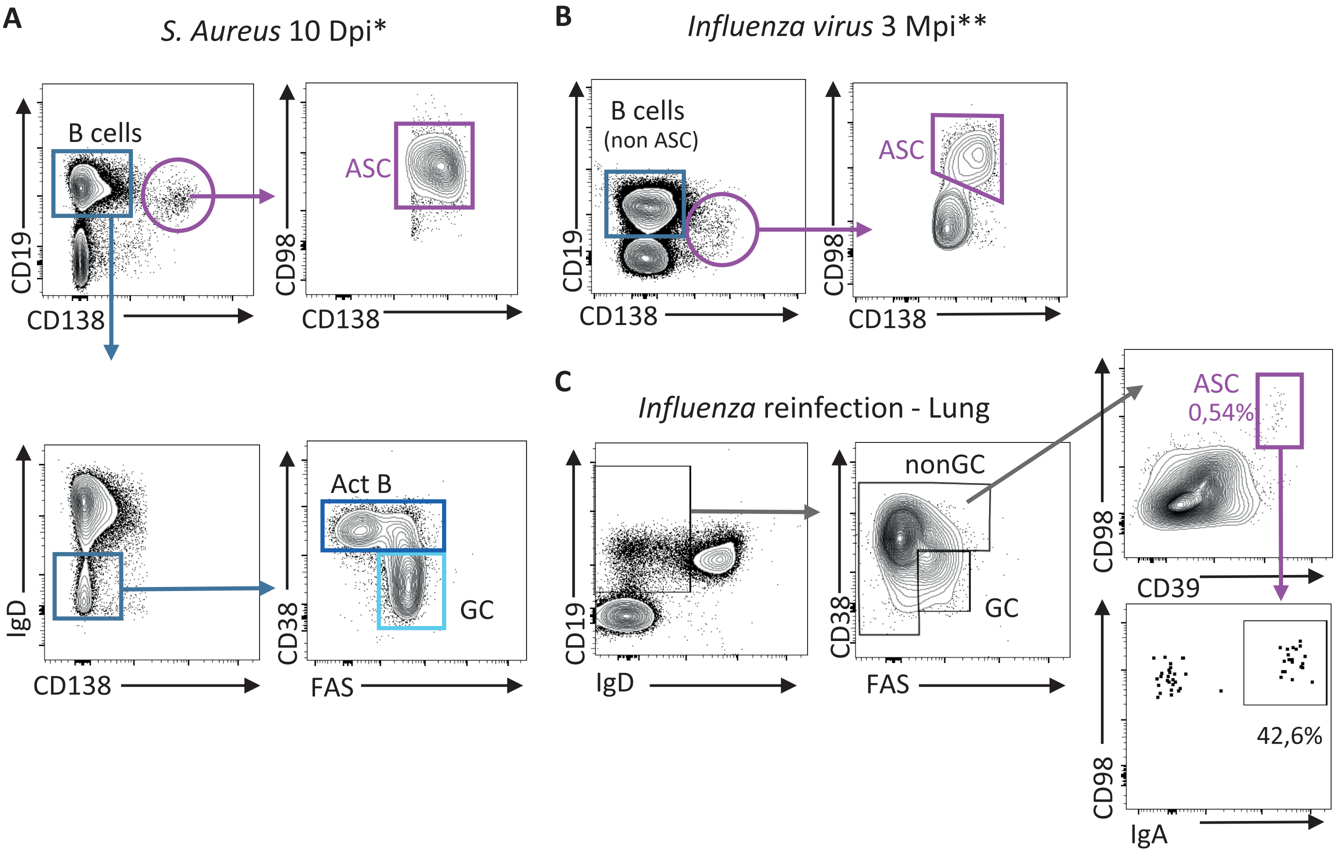

Fig. S2.

Gating strategies for the analysis of B cell subsets and ASC in *S. aureus* and *Influenza* virus-infected mice. (A) Gating strategy to identify activated B (actB) cells (CD138^{neg}CD38^{+}Fas^{neg}B220$^{+)}$, GC B cells (CD138^{neg}CD38^{neg}Fas^{+}B220^{+}), and ASC (CD138^{+}CD98^{+}B220^{int}) in draining LN of 10 Dpi-*S.aureus* infected mice by flow cytometry. *The same gate strategy was used for mediastinal LN from 9 Dpi-Influenza virus-infected mice. **(B)** Gating strategy to identify ASC (CD138^{hi}CD98^{hi}CD19^{int}) and non-ASC (CD138^{neg}CD19$^{+/int}$) in the BM of Influenza virus-infected mice obtained at 3 months post-infection (Mpi). **This gating strategy was also applied to plots from Influenza virus reinfection experiments (for splenic and LN ASC). **(C)** Representative contour plots showing the strategy to identify non-naïve non-GC (IgD^{neg}CD38^{+}Fas^{neg}CD19$^{+)}$ and GC B cells (IgD^{neg}CD38^{neg}Fas^{+} CD19^{+}) and IgA^{+}ASC (IgD^{neg}CD38^{+}Fas^{neg}CD98^{+}CD39^{+}IgA^{+}CD19^{+}) present in lungs from Influenza virus-infected mice, using the CD39, CD98 and IgA. All gates came from singlets, lived lymphocytes. These gate strategies were used for the analysis shown in Fig. 2

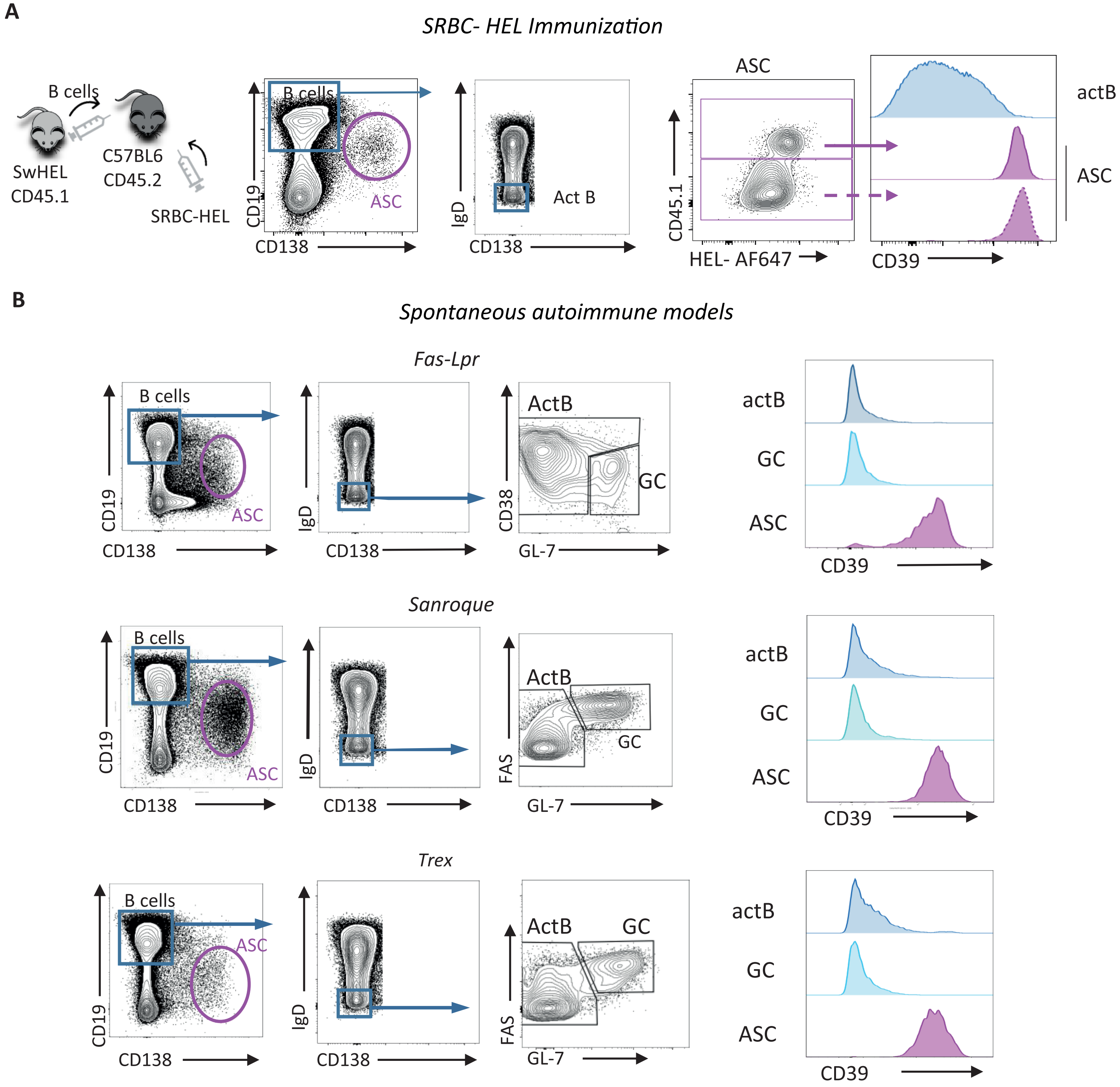

Fig. S3.

ASC generated by immunization or in autoimmune-prone mice expressed high levels of CD39. (A) Schematic representation of B6 mice transferred with purified B cells from MD4 mice CD45.1^{+}, which bear BCR specific for HEL. Subsequently, these mice were immunized with SRBC-HEL. At 14 days post-immunization, the spleens were obtained and the expression of CD39 in ASC (CD138^{+}CD19^{int}) and activated B cells (actB, IgD^{neg}CD19^{+}) was assessed by flow cytometry. Gating strategy showed in contour plots (middle). Representative contour plot of CD45.1 vs HEL expression in ASC, along with histograms depicting CD39 expression in HEL-specific (CD45.1^{+}) and HEL-non-specific (CD45.1^{neg}) ASC. **(B)** Gating strategy to identify ASC (CD138^{hi}CD19^{int}), actB cells (CD138^{neg}IgD^{neg}CD38^{+}GL7^{neg} CD19^{+}), and GC B cells (CD138^{neg}IgD^{neg}CD38^{neg}GL7^{+} CD19^{+}) in the spleen of *FasLpr* mice and to identify ASC (CD138^{hi}CD19^{int}), act B cells (CD138^{neg}IgD^{neg}Fas$^{neg/+}$GL7^{neg}CD19^{+}) and GC B cells (CD138^{neg}IgD^{neg}Fas^{+}GL7^{+} CD19^{+}) in the spleen of *Trex* and *SanRoque* mice, respectively. All gates came from singlets, lived lymphocytes. Representative histograms of CD39 expression in actB, GC, and ASC cells in the spleen of *FasLpr* (n=7), *Sanroque* (n=4), and *Trex* (n=3) mice obtained at 10-12 wk of age. **A** and **B** are representative of two independent experiments with n ≥ 4 each. All gates came from singlets, live lymphocytes.
